# LC–MS-based serum metabolomics analysis for the screening and monitoring of colorectal cancer

**DOI:** 10.3389/fonc.2023.1173424

**Published:** 2023-06-28

**Authors:** Yanan Yi, Jianjian Wang, Chengtong Liang, Chuanli Ren, Xu Lian, Chongxu Han, Wei Sun

**Affiliations:** ^1^ Department of Laboratory Medicine, Northern Jiangsu People’s Hospital Affiliated to Yangzhou University, Yangzhou, Jiangsu, China; ^2^ Institute of Basic Medical Sciences, Chinese Academy of Medical Sciences, School of Basic Medicine, Peking Union Medical College, Beijing, China

**Keywords:** colorectal cancer, serum metabolomics, liquid chromatography-mass spectrometer, biomarkers, screening, monitoring

## Abstract

**Background:**

Colorectal Cancer (CRC) is a prevalent digestive system tumour with significant mortality and recurrence rates. Serum metabolomics, with its high sensitivity and high throughput, has shown potential as a tool to discover biomarkers for clinical screening and monitoring of the CRC patients.

**Methods:**

Serum metabolites of 61 sex and age-matched healthy controls and 62 CRC patients (before and after surgical intervention) were analyzed using a ultra-performance liquid chromatography-high resolution mass spectrometer (UPLC-MS). Statistical methods and pathway enrichment analysis were used to identify potential biomarkers and altered metabolic pathways.

**Results:**

Our analysis revealed a clear distinction in the serum metabolic profile between CRC patients and healthy controls (HCs). Pathway analysis indicated a significant association with arginine biosynthesis, pyrimidine metabolism, pantothenate, and CoA biosynthesis. Univariate and multivariate statistical analysis showed that 9 metabolites had significant diagnostic value for CRC, among them, Guanosine with Area Under the Curve (AUC) values of 0.951 for the training group and0.998 for the validation group. Furthermore, analysis of four specific metabolites (N-Phenylacetylasparticacid, Tyrosyl-Gamma-glutamate, Tyr-Ser and Sphingosine) in serum samples of CRC patients before and after surgery indicated a return to healthy levels after an intervention.

**Conclusion:**

Our results suggest that serum metabolomics may be a valuable tool for the screening and monitoring of CRC patients.

## Introduction

Colorectal cancer (CRC) is currently the third most prevalent malignant tumour and the second leading cause of cancer death worldwide (1).Early detection and treatment are critical in enhancing the 5-year survival rate. Currently, stage I and II patients have a cure rate of approximately 90% (2). Therefore, early screening for CRC is essential for improving patients’ cure and survival rates. Carcinoembryonic antigen (CEA) and faecal occult blood tests are currently the main non-invasive early screening methods, but their clinical value is limited due to their low sensitivity and specificity (3). Endoscopy combined with pathological examination is the gold standard for diagnosing CRC, allowing for an initial evaluation of tumour shape, size,depth of invasion, and pathological classification. However, as an invasive and expensive procedure, it cannot be used for large-scale population screening. Thus, there is an urgent need to develop novel, accurate, and non-invasive techniques for detecting CRC.

Metabolomics is a rapidly developing field that studies the composition, distribution, and regulation of small molecular metabolites. By detecting the metabolic spectrum of biological fluids or tissues and monitoring the effects of different disturbance factors on the body’s metabolic profile (4), it has become a powerful tool for identifying biomarkers for diseases, including cancer. In recent years, metabolomics has been widely applied to the biomarker discovery and pathway analysis of CRC. In 2012, by comparing the area under the receiver operating characteristic curve (AUROC) analysis of 11 amino acids, Leichtle AB et al. found that the model consisting of carcinoembryonic antigen, glycine, and tyrosine had better differentiation for CRC compared to carcinoembryonic antigen alone, with an AUROC of 0.878 (5). Nishiumietal. analyzed serum samples using gas-chromatography/mass-spectrometry (GC/MS) and generated a metabolite panel for CRC detection with an AUC of 0.91 (6). There is an increasing focus on exploring changes in CRC pathways. In 2021, Zhu et al. analyzed the tissue and serum metabonomic profiles of 48 CRC patients using non-targeted GC-MS and found that the most important pathways affecting CRC were phosphate inositol metabolism, primary bile acid biosynthesis, and linoleic acid metabolism pathway (7). Shen et al. applied LC-MS to perform tissue metabolomics for 10 paired CRC tissues and adjacent normal tissues and found alterations in levels of glutathione metabolism, fatty acid metabolism, and amino acid intermediates (8). These studies demonstrate the potential of metabolomics as a promising tool for improving CRC diagnosis and understanding the underlying disease mechanisms.

Metabolomics research of cancer typically relies on biological body fluid samples, withblood and urine being the primary fluids of interest. Blood samples offer a rich source of biological information, with changes in metabolite levels reflecting various pathological changes caused by cancer. Consequently, serum metabolomics has emerged as a promising approach for identifying CRC biomarkers (5, 6). Most studies have focused on identifying markers for the diagnosis of CRC comparing the metabolite profiles of cancer and healthy subjects,demonstrating the potential to distinguish between different stages of cancer based on differential serum metabolite profiles. However, few studies have examined changes in metabolite levels in postoperative patients. Thus, there is an urgent need for further metabolomics research on CRC to fill this gap in knowledge.

In this study, we conducted a non-targeted analysis of human serum using liquid chromatography-high resolution mass spectrometry (LC-HRMS) metabolomics. Specifically, we measured serum metabolites in both newly diagnosed CRC patients and healthy subjects and compared the differential metabolite levels in preoperative and postoperative CRC patients. The objective of this study was to identify potential biomarkers for CRC screening and monitoring and to make a meaningful contribution to clinical research in this area.

## Materials and methods

### Sample collection

All samples were collected from January to August in 2022, according to a standardized sample collection scheme. Specifically, 62 patients with CRC were diagnosed pathologically by the pathology department of Northern Jiangsu People’s Hospital and were not subjected to surgery, chemotherapy, or radiotherapy. The admission criteria of CRC patients included (1): age between 30 and 89 years old (2). clear preoperative diagnosis with complete pathological examination and various examination data (3). absence of other metabolic or immune system diseases, such as diabetes, rheumatoid arthritis, etc. (4) normal laboratory tests including liver function, renal function, and blood routine (5). absence of primary tumours in other parts (6). no prior treatment or medication. Post-operative serum samples were collected one week after the operation.

All samples were collected using a serum collection tube with inert separation gel after an 8-hour overnight fast in the morning. After collection, serum samples were obtained by centrifugation at 3000rpm for 10 minutes and stored at -80 °C for subsequent analysis.

This study was approved by the Ethics Committee of Northern Jiangsu People’s Hospital Affiliated with Yangzhou University (approval number: 2022ky134), and all subjects gave informed consent before participating in this study.

### Sample pre-processing

50 μl serum was mixed with 150 μl acetonitrile and vortexed for 30 seconds, followed by centrifugation at 15,000 × g for 10 minutes. The supernatant was then dried in a vacuum and stored at -80 °C. Before analysis, the dry powder was resuspended in 100 μl 2% acetonitrile, vortexed till it was completely dissolved, and centrifuged at 15,000 × g for 10 minutes. Quality control (QC) samples were combined 5 μl serum samples prepared by mixing the 40 samples randomly from the healthy group, CRC group and the the post-operative group.

### LC-MS/MS analysis

The samples were analysed by Waters ACQUITY H class LC system (Waters, USA) and LTQ Orbitrap Fusion Lumos mass spectrometer (Thermo Fisher, Scientific, MA, USA). The serum metabolites were separated by running a gradient at a flow rate of 0.5 ml/min for 8 minutes on a Waters Acquity UPLC HSS T3 column (100 mm × 3.0 mm, 1.8 μm). The mobile phase A was a 0.1% formic acid aqueous solution, and the mobile phase B was acetonitrile. The gradient elution procedure is as follows: 0-1.0 min, 2%B;1-3 min, 2%-55% B; 3-8min, 55%-100% B.The washing gradient procedure is as follows: 0-3.0 min, 100%B; 3.0-3.1 min, 100-2% B; 3.1-5.0 min, 2% B. The column temperature was set at 40°C, and the injection volume was 20 μl. The electrospray ion source (ESI source), the sheath gas was 40 arb, and the spray voltage was 3.20 kV (positive ion). The range of quality scanning was from 100 to 1000 m/z. The data acquisition mode was set to Full Scan + ddMS2. The goal of MS2 automatic gain control (AGC) was 5 × 10^5^, and the maximum injection time (IT) was 100 ms. High energy collision dissociation (HCD) pyrolysis mode for dissociation has the best collision energy of 20, 35, and 60.

### Data analysis

The original MS data were imported into Progenesis QI (Waters, USA) software for peak alignment, peak picking, and peak recognition. The annotation of metabolites was determined from the accurate mass composition, the isotope goodness-of-fit of the predicted molecular formula and the MS/MS fragments matching with the databases (HMDB, METLIN, and in-house standard libraries). The metabolite score was calculated using the sum of three similarity metrics including mass similarity, isotope similarity, and fragmentation score. The score was used to assess reliability of each metabolite. A CSV file containing sample information, retention time, peak area, score and other data sets was obtained. The CSV file was then imported into MetaboAnalyst5.0 (https://www.metaboanalyst.ca/) for data processing. For following statistical analysis the peak area in each sample were firstly performed normalization to the total compound. Then the missing variables were removed from more than 50% of the samples for further statistical analysis. Student’s t-test was used to evaluate the significance between groups, and α value was set to 0.05. Principal component analysis (PCA) and orthogonal partial least square discriminant analysis (OPLS-DA) were performed using SIMCA14.1 (Umetrics, Sweden) software. The differential metabolites was defined as follows (1): P-value < 0.05 (2); fold change > 1.5 and < 0.67. We used the “pathway analysis” module in MetaboAnalyst 5.0 to analyse the differential metabolites and the “biomarker discovery” module for ROC analysis. In addition, we performed box chart analysis using the R software package (version 3.6.3) to show individual metabolite differences between different groups.

## Results

### Subjects

**Figure 1:**
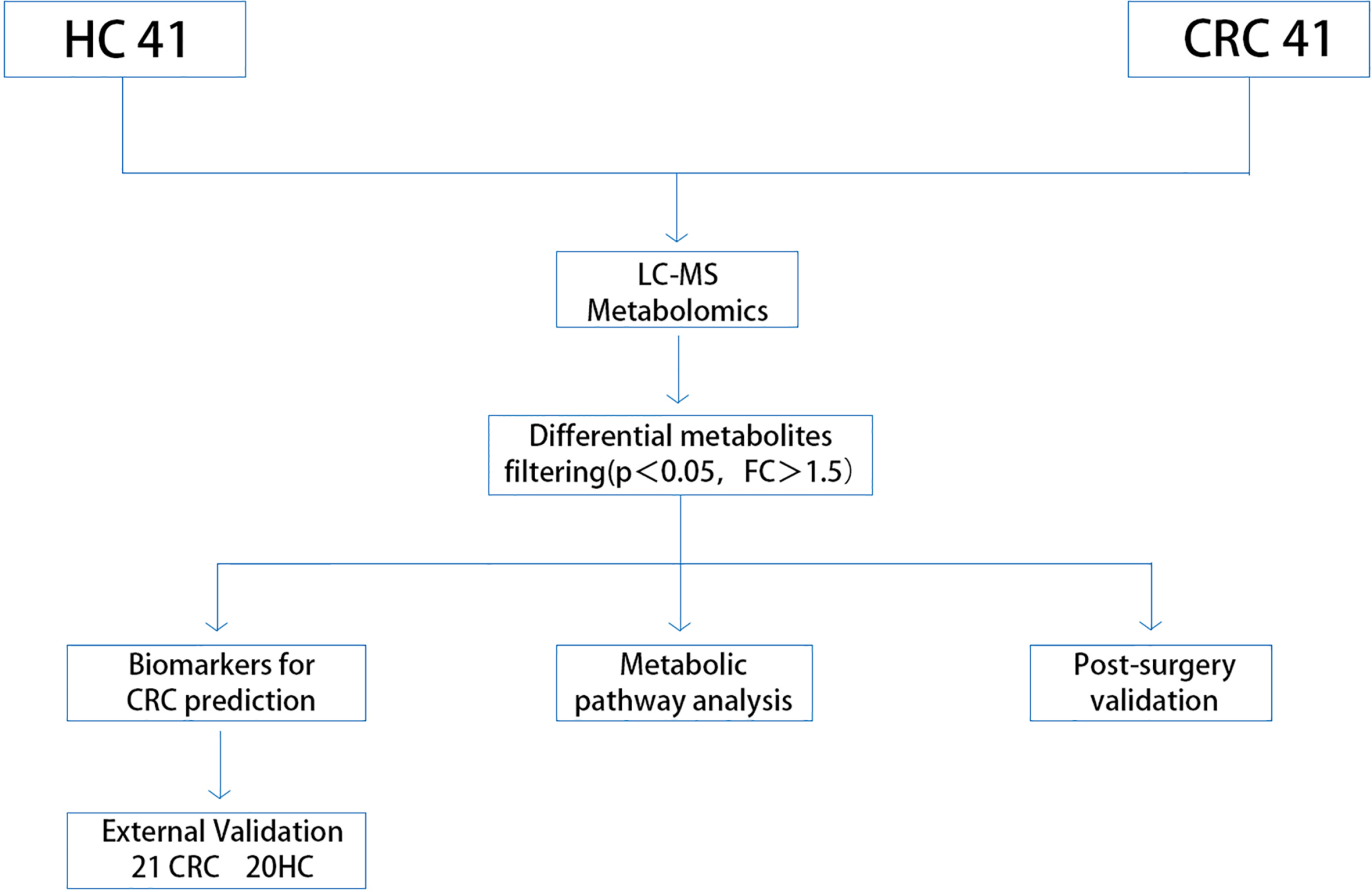
Study workflow. CRC, colorectal cancer; HC, healthy control.

The methodology of this study is illustrated in (**Figure 1**). Our study enrolled a total of 123 subjects, including 61 healthy controls and 62 CRC patients diagnosed pathologically. The samples were randomly divided into a discovery group and a validation group in a 2:1 ratio, with age and sex-matched between the two groups. Differential metabolites were identified through the comparative analysis in 41 age-and sex-matched CRC patients and 41 healthy controls, using a selection criterion of p-value < 0.05 and fold change (FC) >1.5. The identified differential metabolites were subjected to functional annotation and pathway analysis. Moreover, potential biomarkers for CRC diagnosis were discovered through receiver operating characteristic curve (ROC) analysis, which was then validated using an independent set of 21 CRC and 20 healthy control samples. In addition, we collected serum samples from 62 patients one week after the operation and compared the changes in differential metabolites before and after the operation, aiming to identify biomarkers for monitoring prognosis.The details of all study participants are provided in **Table 1** and **Table S1**.

**Table 1:** Basic clinical information of samples.

	Discovery group		Validation group	
	CRC	HC	CRC	HC
Cases	41	41	21	20
Age	66.7 ± 11.0	67.7 ± 7.8	66.4 ± 10.5	61.8 ± 8.3
Sex(M/F)	27/14	27/14	13/9	15/5
Tumour site				
colon	32		14	
rectum	9		7	
AJCC				
I	6		4	
II	15		10	
III	17		7	
IV	3		0	
Lymphatic metastasis	20		7	
Distant metastasis	3		0	

### Quality control

In this study, sample analysis was performed in random order. We evaluated the repeatability of the instrument analysis according to QC correlation. QC sample were randomly run during the sample analysis process. A total of 9 QC samples were injected. The Pearson correlation coefficient analysis was calculated between pairwise pairs of QC results (9). ‘Wu Kong’ platform (https://www.omicsolution.com/wkomics/main/) was used for relative Pearson correlation coefficient analysis of QC samples. The QC chart (**Figure S1**) revealed that the r values (correlation coefficient) were close to 1, indicating the good correlation between QC samples and the LC/MS system stability. This suggested that the observed differences between groups were primarily due to metabolic variations among the samples, rather than any other confounding factors. Moreover, a serum chromatogram of QC sample was provided in **Figure S2**.

### Distinguishing CRC patients from healthy controls using serum metabolomics

**Figure 2:**
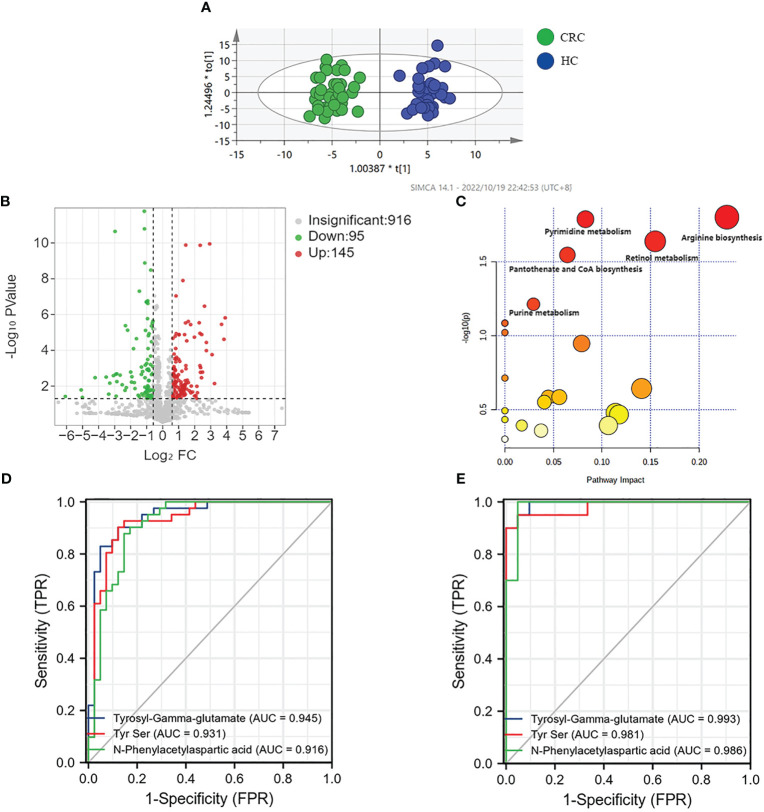
Analysis of metabolic profiling of CRC and HC. **(A)** Metabolic score plot of OPLS-DA. **(B)** Volcano analysis of the metabolites of CRC group and HC group in the discovery group. **(C)** Altered metabolic pathways in colorectal cancer. **(D)** AUC value of three metabolites in the discovery group. **(E)** AUC value of three metabolites in the validation group.

In this study, we conducted unsupervised PCA analysis to identify potential biomarkers distinguishing between CRC patients and healthy controls (**Figure S3A**). We then used supervised pattern recognition with an OPLS-DA model, which showed better separation between the two groups (**Figure 2A**). To ensure the reliability and stability of the supervisory model, we performed 100 permutation tests (**Figure S3B**).

We selected 240 metabolic molecules with statistical differences based on criteria of P-value < 0.05, fold change > 1.5 and < 0.67 (**Table S2**). Of these, 145 metabolites were up-regulated and 95 were down-regulated in the CRC group (**Figure 2B**). Pathway analysis revealed significant disruptions in amino acid metabolism, energy metabolism, and nucleotide metabolism in colon cancer (**Figure 2C**, **Table 2**).We used ROC curve analysis to evaluate the predictive ability of potential biomarkers in distinguishing CRC from healthy controls (**Table S3**). Our results identified 9 metabolites with potential diagnostic value, all of which had AUC higher than 0.9 in the discovery group. In the validation group, the AUC values for these 9 differential metabolites were all above 0.8, indicating good diagnostic value (**Table 3**). Notably, three metabolites- Tyrosyl-Gamma-glutamate, Tyr Ser,and N-Phenylacetylaspartic acid-had AUC values of 0.945, 0.931and 0.916, respectively, in the discovery group (**Figure 2D**), and AUC values of 0.993, 0.981 and 0.986, respectively, in the validation group (**Figure 2E**). Furthermore, mass spectrum of the 9 metabolites were in **Figure S4**.

**Table 2:** Pathway analysis results in the MetaboAnalyst 5.0.

Pathway name	Match status	p	FDR	Impact
Arginine biosynthesis	2/14	0.01579	0.59693	0.22843
Pyrimidine metabolism	3/39	0.016295	0.59693	0.08289
Retinol metabolism	2/17	0.022998	0.59693	0.15464
Pantothenate and CoA biosynthesis	2/19	0.028425	0.59693	0.06429
Purine metabolism	3/65	0.061347	0.98789	0.02939
D-Glutamine and D-glutamate metabolism	1/6	0.082324	0.98789	0

**Table 3:** Differential metabolites for colorectal cancer distinction in the discovery group and validation group.

Compounds	Discovery Group			Validation group		
	AUC Sensitivity Specificity			AUC Sensitivity Specificity		
Guanosine	0.951	0.840	0.913	0.998	0.956	0.958
2-Hydroxyadenine	0.950	0.946	0.856	0.998	0.956	0.958
Tyrosyl-Gamma-glutamate	0.945	0.867	0.878	0.993	0.944	0.958
Tyr Ser	0.931	0.883	0.878	0.981	0.944	0.910
Lyciumoside VI	0.919	0.818	0.832	0.958	1	0.905
3-Hydroxypimelyl-CoA	0.919	0.797	0.889	0.882	0.750	0.952
N-Phenylacetylaspartic acid	0.916	0.892	0.821	0.986	1	0.952
Sphingosine	0.914	0.878	0.818	0.843	0.789	0.836
Val Arg	0.908	0.805	0.878	0.840	0.878	0.683

### Discovery of metabolic markers for postoperative monitoring of CRC

**Figure 3:**
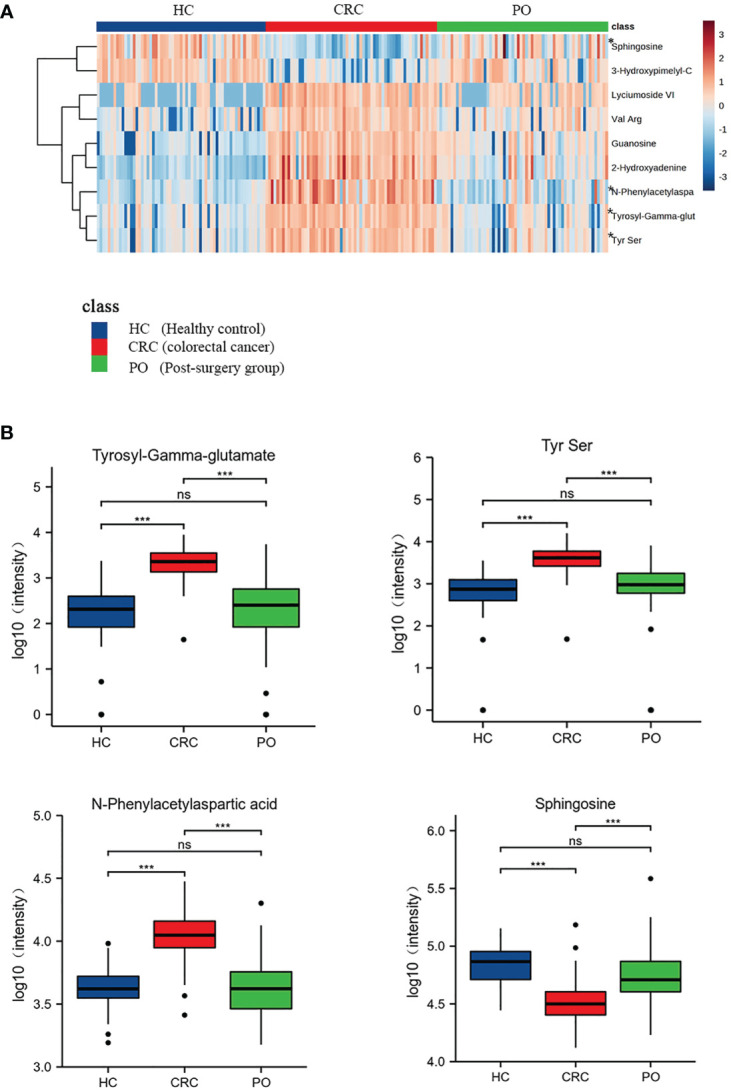
Heat map of 9 CRC-related differential metabolites in CRC,healthy control and post-surgery group. “*” marked the four metabolites which returned to normal levels **(A)**. The box plots of these 4 metabolites of healthy controls, CRC group and the post-surgery group **(B)**. “ns” p >0.05; “***”p < 0.001.

In this study, postoperative specimens from CRC patients were analyzed to evaluate the association of the identified differential metabolites with tumour load. These metabolites may serve as potential biomarkers for monitoring CRC after surgery. Therefore, we collected serum samples about a week after the operation from 62 cases and examined the changing trend of the 9 metabolites identified earlier in preoperative and postoperative cases to evaluate the biological correlation between the potential biomarkers and CRC tumour load. We analyzed the mean intensity heatmap (**Figure 3A**) and serum levels of the 9 specific metabolites before and after the operation (**Figure 3B**, **Figure S5**, **Table 4**).

**Table 4:** The intensity changes of differential metabolites before and after operation.

Compounds	HC		CRC		PO	
	Mean	SD	Mean	SD	Mean	SD
Guanosine	557.31	763.49	8005.65	8798.36	2462.23	3680.06
2-Hydroxyadenine	923.64	602.04	5929.21	5268.45	2405.23	2514.34
Tyrosyl-Gamma-glutamate*	306.35	397.89	2589.12	1772.56	548.71	934.24
Tyr Ser*	945.63	809.55	4695.36	2886.14	1388.29	1526.21
Lyciumoside VI	1664.32	3882.26	29353.63	37950.91	6065.67	7345.01
3-Hydroxypimelyl-CoA	52332.62	35776.92	7681.90	8146.51	40996.07	44496.98
N-Phenylacetylaspartic acid*	4490.92	1612.21	12460.65	5980.75	4975.19	3143.40
Sphingosine*	72805.68	27915.05	36202.03	21451.68	67715.09	52913.39
Val Arg	1484.90	2532.48	6160.23	6194.21	2523.44	3256.98

CRC, colorectal cancer; HC, healthy control; PO, post-surgery group.”*” refer to four metabolites which returned to normal levels.

The intensity heatmap showed that seven metabolites were remarkably up-regulated in CRC and decreased after operation. These metabolites were related to amino acid metabolism and purine metabolism. Several studies have reported that the up-regulation of amino acids and purine metabolism was to promote the proliferation of cancer cells (10, 11). And the rest two down-regulated metabolites, 3-Hydroxypimelyl-CoA and Sphingosine, were involved in lipid metabolism. Lipid metabolism was associated with tumor progression and metastasis, which was to maintain high energy demand and division of cancer cells (12, 13). Moreover, previous research reported that the changes of lipidomic signatures could be served as promising potential biomarkers (12, 14).

The results showed significant differences in the levels of the 9 specific metabolites in preoperative and postoperative cases. Among these, 3-Hydroxypimelyl-CoA and Sphingosine were higher in healthy controls than in CRC, while Guanosine, 2-Hydroxyadenine, Tyrosyl-Gamma-glutamate, Tyrs Ser, Lyciumoside VI, N-Phenylacetylasparticacid, and Val Arg were higher in CRC. Four of the 9 specific metabolites, including N-Phenylacetylasparticacid, Tyrosyl-Gamma-glutamate,Tyr Ser and Sphingosine returned to normal levels, and there was no significant difference between post-operation and healthy controls (**Figure 3B**). These metabolites may be related to tumour load and can be used to monitor the treatment of CRC after surgery. These results further confirm the biological correlation of these metabolites in CRC and highlight their potential value for the screening and monitoringof CRC. The remaining five metabolites exhibited statistical differences between preoperative CRC samples and healthy controls and tended to return to normal levels after the operation. However, further validation may be required to confirm this (**Figure S5**).

Moreover, we explored whether these 9 metabolites were statistically significant in different genders, tumor locations, AJCC stage and TNM classification. There was no statistical significance among different groups (**Table S4**). Only Val Arg was statistically different (P<0.05) in the comparison of left and right-sided colorectal cancer. However, the specific mechanism is still unclear and needs to be further investigated.

## Discussion

Serum metabolomics analysis using mass spectrometry offers high-throughput and high-sensitivity advantages for the screening of CRC. In this study, we comprehensively characterized the serum metabolomic profiles of healthy controls, preoperative CRC patients andpostoperative CRC patients. We identified 9specific differential metabolites that exhibit good discriminatory power for distinguishing CRC patients from healthy controls. Moreover, the 9 metabolites exhibited significant differences between preoperative and one-week post-operative samples, with four metabolites including N-Phenylacetylasparticacid, Tyrosyl-Gamma-glutamate, Tyr Ser and Sphingosine returning to normal levels and displaying no significant difference compared to healthy controls after surgery. Thus, these four metabolites may serve as potential biomarkers for monitoring CRC.

In this study, we observed changes in several metabolic pathways, including arginine biosynthesis, purine metabolism, and pantothenate and CoA biosynthesisin patients with CRC group. These metabolic changes are typical of tumour cells, which must adapt to the nutrition-deficient environment, and obtain the necessary nutrients to support their rapid proliferation and the establishment of a new biological microenvironment (10). Our findings indicated that Guanosine, an intermediate metabolite in the purine pathway and a common precursor of DNA, was significantly increased in the CRC group compared to the control group. This up-regulated metabolite change suggested that purine metabolism was upregulated in CRC. Consistent with our results, previous metabolomics analyses of CRC tissues have reported upregulation of urea cycle intermediates, purines, and most amino acids (15). Abnormal proliferation of cancer cells is one of the hallmarks of cancer, and purine is one of the basic nucleotides needed for cell proliferation, underscoring the close relationship between purine metabolism and cancer (11). Furthermore, increased purine levels are also considered an indicator of enhanced DNA synthesis (16). In 2017, Tian et al. examined the expression level of a rate-limiting enzyme gene in the purine synthesis pathway in different cancers and found that purine metabolism was significantly upregulated in colorectal adenocarcinoma, bladder cancer, breast cancer, and other cancers (17). The decrease in guanosine levels after surgery in our study further confirmed that solid tumour resection led to the cessation of abnormal cancer cell proliferation and that purine metabolism gradually returned to normal.

Amino acids are known to be essential for cell proliferation, and certain cancer cells rely on specific amino acids as their primary energy sources (18). Hirayama A et al. conducted a study comparing the metabolites of colon cancer and normal tissues and observed significantly higher levels of most amino acids and their primary derivatives in tumours compared to normal colon tissues (10). Arginine biosynthesis, a critical pathway upregulated in CRC compared with healthy controls, is of particular interest. Two intermediates involved in this pathway, L-citrulline and L-glutamine were found to exhibit contrasting changes in the CRC group. While L-citrulline decreased, L-glutamine increased in the CRC group. L-citrulline is mainly produced in the small intestine and converted to arginine through the actions of arginine succinate synthase (ASS) and arginine succinate lyase (ASL) (19). Glutamine, on the other hand, is the most abundant free amino acid in serum (20), and serves as an essential energy source for cancer cell proliferation (21). It also acts as a nitrogen donor, necessary for *de novo* synthesis of purines and pyrimidines, promoting nucleotide production during cancer cell proliferation (22). Within cells,glutamine is synthesized from glutamate and ammonia by glutamine synthetase (Glutamine synthetase, GS), which is highly expressed in hepatocellular carcinoma (HCC) and glioblastoma (23, 24). Extracellular glutamine can act as a signal transducer, activating transcriptional activator 3 (STAT3) to promote cancer cell proliferation (21). Arginine is one of the essential amino acids in the human body, and it plays a vital role in the ornithine cycle and promotes the formation of urea. The ammonia produced in the human body is converted into urea through the ornithine cycle and excreted through the urine. Arginine is the precursor of nitric oxide (NO) synthesis, and NO is an important signal molecule involved in immune and vascular tone regulation (25). Previous studies have shown that the potential link between arginine and colorectal cancer is the regulation of the immune system by arginine through nitric oxide (25, 26). Whereas low concentrations of NO amplify the Ras signal by inducing conformational changes of membrane-bound Ras protein to promote cancer cell proliferation (27). high concentrations of NO may lead to apoptosis, invasion, and metastasis (28). Catabolic disease states (such as sepsis, injury, and cancer) can lead to increased arginine utilization, resulting in increased arginine synthesis. Arginine is the precursor of proline, which is necessary for the synthesis of collagen. Moreover, it produces polyamines under the action of ornithine decarboxylase to promote the occurrence of CRC and cell proliferation (29, 30). As the arginine metabolic pathway is highly active in colorectal cancer, multiple molecules or enzymes involved in this pathway may be promising targets for targeted therapy for colorectal cancer (31). It has been found that tumour-infiltrating dendritic cells inhibit the proliferation and activation of CD8 cells through L-arginine metabolism (32). In addition, the expression of enzymes involved in arginine metabolism is increased in CRC tumour cells, and increasing research is focusing on potential ways to interfere with the regulatory mechanism of the L-arginine pathway by targeting transporters (10).

In this study, we also observed disruption of the pantothenate and CoA biosynthesis pathways in the CRC group. Currently, there are very few reports on the status of pantothenate and CoA biosynthesis pathways in cancer. Pantothenate, also known as vitamin B5, is one of the components of coenzyme A. Coenzyme A(CoA) plays a key role in energy and lipid synthesis (16). The increased concentration of coenzyme A in the body promotes the transition from glucose oxidation to fatty acid oxidation, thus stimulating gluconeogenesis (33). Recent studies have found that TC22 cells in CD8+-effector T cells highly express the pantothenate-CoA pathway, and CoA enhances the anti-tumour ability of TC22 by promoting oxidative phosphorylation (34, 35). These results suggest that the disruption of this pathway may be partly caused by the limited energy supply and deficiency of anti-tumour effector T cells in patients with CRC.

Tyrosyl-Gamma-glutamate is a dipeptide synthesized by tyrosine and γ-glutamate, and was significantly increased in the CRC group (AUC>0.9 in both the discovery group and validation group) and returned to normal levels after the operation. Therefore, it can have utility as a metabolic marker for detection and postoperative monitoring. When tyrosine is phosphorylated by tyrosine kinase, it regulates the signal transduction pathway and activates pyruvate dehydrogenase kinase 1 (PDHK1), which promotes solid tumour growth and Warburg metabolism (36, 37). Glutamine is deaminated to glutamic acid under the catalysis of glutaminase (GLS), which is then deaminated by glutamate dehydrogenase (GDH) to form α-ketoglutarate, which enters the TCA cycle and serves as a precursor for the synthesis of certain amino acids (38). In a previous study, analysis of levels of intracellular ROS in different cancers revealed a strong positive correlation between the estimated change in ROS levels in cancer and the change in levels of glutamate metabolism (r = 0.655, P = 0.029) (17). This may be related to the synthesis of glutathione by glutamate (39). Previous studies found that the increase of glutamate and glutathione levels is an important signal for oxidative stress, which may be due to the metabolism of rapidly proliferating CRC cells and glutathione metabolism is upregulated to combat the oxidative stress (8). Tyrosine and γ-glutamate, which are involved in dipeptide synthesis, are directly or indirectly implicated in colorectal tumours. The increased protein catabolism in CRC patients results in elevated levels of Tyrosyl-Gamma-glutamate in the serum. The observed decrease in the levels of Tyrosyl-Gamma-glutamate after the surgical removal of the tumour may be due to the decrease in energy metabolism and oxidative stress, which could result in a gradual shift toward normal levels of protein metabolism. However, currently, there is a lack of literature on the regulation/transformation mechanism of dipeptides in colorectal tissues.

N-Phenylacetylaspartic acid belongs to a class of aspartic acidderivatives. Aspartic acid is produced by oxaloacetic acid, an intermediate product involved in the tricarboxylic acid cycle (TCA). TCA disorders are related to the occurrence and development of colon cancer (10, 16). Aspartic acid also reacts with citrulline to form arginine, which enters the urea cycle. In this study, we found that the level of serum N-Phenylacetylasparticacid in CRC patients was higher than that in healthy controls. This may be because aspartic acid is also utilized by cells for nucleotide biosynthesis, which is very important for cancer cell proliferation and is often up-regulated in tumors (40). Through comparing metabolite profiles at various stages of CRC, previous studies have found that serum aspartic acid and other amino acids peak significantly enhanced in patients with stage 3-4 CRC (6). Consistent with our findings,the level of N-Phenylacetylasparticacid decreases after the operation, which can be attributed to the decrease or absence of cancer cell proliferation, which would bring nucleotide metabolism back to normal. Therefore, N-Phenylacetylasparticacid may be an effective biomarker for monitoring patients’ metabolism patterns before and after surgery.

Our results confirmed that Sphingosine reduced in the CRC group and returned to normal levels in the post-operative group. Sphingosine is the major component of sphingolipids, which belongs to cell membrane lipids. Phosphorylate Sphingosine forms the bioactive lipid sphingosine 1-phosphate (S1P), catalyzed by sphingosine kinase1 (Sphk1) (41). Several studies have revealed that the SIP/Sphk1 signaling plays oncogenic roles and it is overexpressed in colon cancer tissue which is correlated with poor survival (41–43). Therefore, decreased Sphingosine levels in CRC group may be due to increased utilization of lipids or enhanced SIP synthesis, which is needed for increased membrane synthesis or tumour development.

## Conclusion

In conclusion, we performed LC-MS-based comparative metabolomics to evaluate the serum metabolite profiles of healthy controls and preoperative and postoperative CRC patients. 9 metabolites were identified as potential biomarkers of CRC. Of these, N-Phenylacetylasparticacid, Tyrosyl-Gamma-glutamate, Tyr-Ser and Sphingosine, showed similar levels in healthy controls and post-operative CRC patients, which indicates their potential value in screening and postoperative monitoring of CRC.

Nonetheless, further detailed studies are required to validate our findings. There are several ways in which this could be achieved. First, in this pilot study, we tentatively explored CRC-associated serum metabolite changes and potential biomarkers. We provided some clues for functional analysis and subsequent study. Due to the small sample size, our analysis is preliminary. In the future, larger sample cohorts from multicenter analyses should be analyzed and standards validation will be necessary. Second, a grouping study of inflammatory bowel disease should be included, which could reveal deeper mechanisms of CRC development. Third, patients with infections and metabolic diseases were not enrolled in this study, which may limit the application of our conclusions. We will include these patients in future analysis for a more comprehensive validation. Forth, in this study, we collected post-operative serum samples only once, one week after the operation. However, we did not subsequently perform a follow-up analysis on postoperative patient samples.Therefore, future studies could include an analysis of samples collected over longer periods after the surgery, which could be useful in dynamically monitoring the preoperative and postoperative metabolic changes. Additionally, among the 9 potential biomarkers, three did not have standard secondary mass spectra. Further studies will be done for the validation of the 3 metabolites.

## Data availability statement

The datasets presented in this study can be found in online repositories. The names of the repository/repositories and accession number(s) can be found in the article/**Supplementary Material**.

## Ethics statement

The studies involving human participants were reviewed and approved by Ethics Committee of Northern Jiangsu People’s Hospital Affiliated with Yangzhou University (approval number: 2022ky134). The patients/participants provided their written informed consent to participate in this study. Written informed consent was obtained from the individual(s) for the publication of any potentially identifiable images or data included in this article.

## Author contributions

YY and WS designed the study, performed data processing and statistical analysis. XL collected the serum samples. The article was written by YY. CL and CR contributed to the final version of the manuscript. CH and WS participated in generation of the manuscript and are the corresponding authors. All authors contributed to the article and approved the submitted version.

## References

[1] Sung H Ferlay J Siegel RL Laversanne M Soerjomataram I Jemal A. Global cancer statistics 2020: GLOBOCAN estimates of incidence and mortality worldwide for 36 cancers in 185 countries. CA Cancer J Clin (2021) 71(3):209–49. doi: 10.3322/caac.21660 33538338

[2] Weitz J Koch M Debus J Höhler T Galle PR Büchler MW. Colorectal cancer. Lancet (2005) 365(9454):153–65. doi: 10.1016/S0140-6736(05)17706-X 15639298

[3] Chan EC Koh PK Mal M Cheah PY Eu KW Backshall A. Metabolic profiling of human colorectal cancer using high-resolution magic angle spinning nuclear magnetic resonance (HR-MAS NMR) spectroscopy and gas chromatography mass spectrometry (GC/MS). J Proteome Res (2009) 8(1):352–61. doi: 10.1021/pr8006232 19063642

[4] Kikuchi K Kakeya H. A bridge between chemistry and biology. Nat Chem Biol (2006) 2(8):392–4. doi: 10.1038/nchembio0806-392 16850010

[5] Leichtle AB Nuoffer JM Ceglarek U Kase J Conrad T Witzigmann H. Serum amino acid profiles and their alterations in colorectal cancer. Metabolomics (2012) 8(4):643–53. doi: 10.1007/s11306-011-0357-5 PMC3397217 22833708

[6] Nishiumi S Kobayashi T Ikeda A Yoshie T Kibi M Izumi Y. A novel serum metabolomics-based diagnostic approach for colorectal cancer. PloS One (2012) 7(7):e40459. doi: 10.1371/journal.pone.0040459 22792336 PMC3394708

[7] Zhu G Wang Y Wang W Shang F Pei B Zhao Y. Untargeted GC-MS-Based metabolomics for early detection of colorectal cancer. Front Oncol (2021) 11:729512. doi: 10.3389/fonc.2021.729512 34804922 PMC8599589

[8] Shen Y Sun M Zhu J Wei M Li H Zhao P. Tissue metabolic profiling reveals major metabolic alteration in colorectal cancer. Mol Omics. (2021) 17(3):464–71. doi: 10.1039/D1MO00022E 33881127

[9] Wang S Zhu H Zhou H Cheng J Yang H. MSpectraAI: a powerful platform for deciphering proteome profiling of multi-tumor mass spectrometry data by using deep neural networks. BMC Bioinf (2020) 21:439. doi: 10.1186/s12859-020-03783-0 PMC7539376 33028193

[10] Hirayama A Kami K Sugimoto M Sugawara M Toki N Onozuka H. Quantitative metabolome profiling of colon and stomach cancer microenvironment by capillary electrophoresis time-of-flight mass spectrometry. Cancer Res (2009) 69(11):4918–25. doi: 10.1158/0008-5472.CAN-08-4806 19458066

[11] Yin J Ren W Huang X Deng J Li T Yin Y. Potential mechanisms connecting purine metabolism and cancer therapy. Front Immunol (2018) 9:1697. doi: 10.3389/fimmu.2018.01697 30105018 PMC6077182

[12] Mozolewska P Duzowska K Pakiet A Mika A ŚledziŃski T. Inhibitors of fatty acid synthesis and oxidation as potential anticancer agents in colorectal cancer treatment. Anticancer Res (2020) 40:4843–56. doi: 10.21873/anticanres.14487 32878772

[13] Răchieriu C Eniu DT Moiş E Graur F Socaciu C Socaciu MA. Lipidomic signatures for colorectal cancer diagnosis and progression using UPLC-QTOF-ESI(+)MS. Biomolecules (2021) 11(3):417. doi: 10.3390/biom11030417 33799830 PMC8035671

[14] Coleman O Ecker M Haller D. Dysregulated lipid metabolism in colorectal cancer. Curr Opin Gastroenterol (2022) 38:162–7. doi: 10.1097/MOG.0000000000000811 35098938

[15] Denkert C Budczies J Weichert W Wohlgemuth G Scholz M Kind T. Metabolite profiling of human colon carcinoma–deregulation of TCA cycle and amino acid turnover. Mol Cancer. (2008) 7:72. doi: 10.1186/1476-4598-7-72 18799019 PMC2569965

[16] Naquet P Kerr EW Vickers SD Leonardi R. Regulation of coenzyme a levels by degradation: the 'Ins and outs'. Prog Lipid Res (2020) 78:101028. doi: 10.1016/j.plipres.2020.101028 32234503 PMC7234920

[17] Tian Y Du W Cao S Wu Y Dong N Wang Y. Systematic analyses of glutamine and glutamate metabolisms across different cancer types. Chin J Cancer. (2017) 36(1):88. doi: 10.1186/s40880-017-0255-y 29116024 PMC5678792

[18] Argilés JM Azcón-Bieto J. The metabolic environment of cancer. Mol Cell Biochem (1988) 81(1):3–17. doi: 10.1007/BF00225648 3050448

[19] Morris SM Jr. Arginine metabolism revisited. J Nutr (2016) 146(12):2579S–86S. doi: 10.3945/jn.115.226621 27934648

[20] Brosnan JT. Interorgan amino acid transport and its regulation. J Nutr (2003) 133(6 Suppl 1):2068S–72S. doi: 10.1093/jn/133.6.2068S 12771367

[21] Cacace A Sboarina M Vazeille T Sonveaux P. Glutamine activates STAT3 to control cancer cell proliferation independently of glutamine metabolism. Oncogene (2017) 36(15):2074–84. doi: 10.1038/onc.2016.364 PMC5245769 27748760

[22] DeBerardinis RJ Cheng T. Q's next: the diverse functions of glutamine in metabolism, cell biology and cancer. Oncogene (2010) 29(3):313–24. doi: 10.1038/onc.2009.358 PMC2809806 19881548

[23] Long J Lang ZW Wang HG Wang TL Wang BE Liu SQ. Glutamine synthetase as an early marker for hepatocellular carcinoma based on proteomic analysis of resected small hepatocellular carcinomas. Hepatobiliary Pancreat Dis Int (2010) 9(3):296–305. 20525558

[24] Rosati A Poliani PL Todeschini A Cominelli M Medicina D Cenzato M. Glutamine synthetase expression as a valuable marker of epilepsy and longer survival in newly diagnosed glioblastoma multiforme. Neuro Oncol (2013) 15(5):618–25. doi: 10.1093/neuonc/nos338 PMC3635515 23410662

[25] Tong BC Barbul A. Cellular and physiological effects of arginine. Mini Rev Med Chem (2004) 4(8):823–32. doi: 10.2174/1389557043403305 15544543

[26] Ma Q Wang Y Gao X Ma Z Song Z. L-arginine reduces cell proliferation and ornithine decarboxylase activity in patients with colorectal adenoma and adenocarcinoma. Clin Cancer Res (2007) 13(24):7407–12. doi: 10.1158/1078-0432.CCR-07-0751 18094424

[27] Pervin S Singh R Hernandez E Wu G Chaudhuri G. Nitric oxide in physiologic concentrations targets the translational machinery to increase the proliferation of human breast cancer cells: involvement of mammalian target of rapamycin/eIF4E pathway. Cancer Res (2007) 67(1):289–99. doi: 10.1158/0008-5472.CAN-05-4623 17210710

[28] Choudhari SK Chaudhary M Bagde S Gadbail AR Joshi V. Nitric oxide and cancer: a review. World J Surg Oncol (2013) 11:118. doi: 10.1186/1477-7819-11-118 23718886 PMC3669621

[29] Witte MB Barbul A. Arginine physiology and its implication for wound healing. Wound Repair Regen. (2003) 11(6):419–23. doi: 10.1046/j.1524-475X.2003.11605.x 14617280

[30] Gerner EW Meyskens FL Jr. Combination chemoprevention for colon cancer targeting polyamine synthesis and inflammation. Clin Cancer Res (2009) 15(3):758–61. doi: 10.1158/1078-0432.CCR-08-2235 PMC2666541 19188144

[31] Uchiyama K Yagi N Mizushima K Higashimura Y Hirai Y Okayama T. Serum metabolomics analysis for early detection of colorectal cancer. J Gastroenterol (2017) 52(6):677–94. doi: 10.1007/s00535-016-1261-6 27650200

[32] Norian LA Rodriguez PC O'Mara LA Zabaleta J Ochoa AC Cella M. Tumor-infiltrating regulatory dendritic cells inhibit CD8+ T cell function *via* l-arginine metabolism. Cancer Res (2009) 69(7):3086–94. doi: 10.1158/0008-5472.CAN-08-2826 PMC2848068 19293186

[33] Leonardi R Rehg JE Rock CO Jackowski S. Pantothenate kinase 1 is required to support the metabolic transition from the fed to the fasted state. PloS One (2010) 5(6):e11107. doi: 10.1371/journal.pone.0011107 20559429 PMC2885419

[34] St Paul M Saibil SD Han S Israni-Winger K Lien SC Laister RC. Coenzyme a fuels T cell anti-tumor immunity. Cell Metab (2021) 33(12):2415–2427.e6. doi: 10.1016/j.cmet.2021.11.010 34879240

[35] Tabata R Chi S Yuda J Minami Y. Emerging immunotherapy for acute myeloid leukemia. Int J Mol Sci (2021) 22(4):1944. doi: 10.3390/ijms22041944 33669431 PMC7920435

[36] Hitosugi T Fan J Chung TW Lythgoe K Wang X Xie J. Tyrosine phosphorylation of mitochondrial pyruvate dehydrogenase kinase 1 is important for cancer metabolism. Mol Cell (2011) 44(6):864–77. doi: 10.1016/j.molcel.2011.10.015 PMC3246218 22195962

[37] Taddei ML Pardella E Pranzini E Raugei G Paoli P. Role of tyrosine phosphorylation in modulating cancer cell metabolism. Biochim Biophys Acta Rev Cancer. (2020) 1874(2):188442. doi: 10.1016/j.bbcan.2020.188442 33017632

[38] Lu W Pelicano H Huang P. Cancer metabolism: is glutamine sweeter than glucose. Cancer Cell (2010) 18(3):199–200. doi: 10.1016/j.ccr.2010.08.017 20832746 PMC2952340

[39] Traverso N Ricciarelli R Nitti M Marengo B Furfaro AL Pronzato MA. Role of glutathione in cancer progression and chemoresistance. Oxid Med Cell Longev (2013) 2013:972913. doi: 10.1155/2013/972913 23766865 PMC3673338

[40] Shuvalov O Petukhov A Daks A Fedorova O Vasileva E Barlev NA. One-carbon metabolism and nucleotide biosynthesis as attractive targets for anticancer therapy. Oncotarget (2017) 8(14):23955–77. doi: 10.18632/oncotarget.15053 PMC5410357 28177894

[41] Bao Y Guo Y Zhang C Fan F Yang W. Sphingosine kinase 1 and sphingosine-1-Phosphate signaling in colorectal cancer. Int J Mol Sci (2017) 18(10):2109. doi: 10.3390/ijms18102109 28991193 PMC5666791

[42] Olivera A Kohama T Edsall L Nava V Cuvillier O Poulton S. Sphingosine kinase expression increases intracellular sphingosine-1-phosphate and promotes cell growth and survival. J Cell Biol (1999) 147:545–58. doi: 10.1083/jcb.147.3.545 PMC2151183 10545499

[43] Liu SQ Su YJ Qin MB Mao YB Huang JA Tang GD. Sphingosine kinase 1 promotes tumor progression and confers malignancy phenotypes of colon cancer by regulating the focal adhesion kinase pathway and adhesion molecules. Int J Oncol (2013) 42:617–26. doi: 10.3892/ijo.2012.1733 23232649

