# Supplementary material for: LC–MS-based serum metabolomics analysis for the screening and monitoring of colorectal cancer
Source: Front Oncol. 2023 Jun 28;13:1173424. doi: 10.3389/fonc.2023.1173424 (PMC10338013; doi:10.3389/fonc.2023.1173424)
Supplement: Supplementary file 1 [file Table_1.docx]

***Supplementary Tables***

**Table S1** Detailed patients information

| **Discovery Group** | | | | |  |
| --- | --- | --- | --- | --- | --- |
| **ID** | **Sex** | **Age** | **Tumor sites** | **TNM** | **AJCC** |
| 2 | M | 74 | sigmoid | T4N0M0 | II |
| 9 | F | 42 | sigmoid | T3N1M0 | III |
| 30 | M | 64 | sigmoid | T4N1M0 | III |
| 34 | M | 70 | sigmoid | T4N2M1 | IV |
| 36 | M | 84 | ascending colon | T3N1M0 | III |
| 40 | M | 54 | descending colon | T3N0M0 | II |
| 86 | M | 79 | rectum | T3N0M0 | II |
| 118 | M | 67 | sigmoid | T3N2M0 | III |
| 121 | M | 53 | hepatic flexure | T4N0M0 | II |
| 123 | F | 56 | sigmoid | T1N0M0 | I |
| 126 | M | 75 | sigmoid | T4N0M0 | II |
| 129 | M | 69 | descending colon | T3N1M0 | III |
| 131 | M | 79 | transverse colon | T4N0M0 | II |
| 132 | F | 57 | ascending colon | T4N2M0 | III |
| 133 | F | 49 | sigmoid | T3N1M0 | III |
| 135 | F | 71 | ascending colon | T3N2M0 | III |
| 138 | M | 62 | descending colon | T3N0M0 | II |
| 139 | M | 75 | sigmoid | T4N1M0 | III |
| 140 | F | 55 | sigmoid | T3N0M0 | II |
| 144 | M | 75 | ascending colon | T4N2M1 | IV |
| 145 | M | 81 | transverse colon | T4N2M0 | III |
| 146 | M | 50 | sigmoid | T3N0M0 | II |
| 148 | M | 73 | sigmoid | T1N0M0 | I |
| 149 | M | 77 | rectum | T2N1M0 | III |
| 151 | F | 72 | rectum | T1N0M0 | I |
| 152 | F | 65 | sigmoid | T3N0M0 | II |
| 153 | F | 59 | sigmoid | T3N0M0 | II |
| 155 | M | 65 | rectum | T3N1M0 | III |
| 156 | M | 68 | ascending colon | T3N0M0 | II |
| 157 | M | 50 | rectum | T3N0M0 | II |
| 158 | F | 75 | sigmoid | T2N0M0 | I |
| 159 | F | 85 | sigmoid | T4N2M0 | III |
| 160 | M | 57 | rectum | T1N0M0 | I |
| 161 | M | 68 | rectum | T3N2M0 | III |
| 162 | M | 66 | rectum | T4N2M1 | IV |
| 168 | M | 73 | rectum | T4N0M0 | II |
| 171 | F | 80 | ascending colon | T3N1M0 | III |
| 172 | M | 83 | descending colon | T3N1M0 | III |
| 174 | F | 65 | rectum | T1N0M0 | I |
| 175 | F | 50 | sigmoid | T2N1M0 | III |
| 176 | M | 63 | sigmoid | T4N0M0 | II |
| **Validation Group** | | | | |  |
| **ID** | **Sex** | **Age** | **Position** | **TNM** |  |
| 177 | F | 73 | colon | T2N0M0 | I |
| 179 | F | 55 | rectum | T2N0M0 | I |
| 180 | F | 55 | rectum | T2N1M0 | III |
| 182 | M | 65 | sigmoid | T3N0M0 | II |
| 184 | M | 69 | rectum | T4N0M0 | II |
| 185 | F | 67 | rectum | T4N0M0 | II |
| 187 | F | 46 | sigmoid | T3N2M0 | III |
| 188 | M | 78 | right hemicolon | T4M0M0 | II |
| 189 | M | 58 | ascending colon | T3N0M0 | II |
| 191 | F | 80 | sigmoid | T3N0M0 | II |
| 192 | M | 75 | rectum | T3N1M0 | III |
| 193 | M | 45 | sigmoid | TisN0M0 | I |
| 194 | M | 65 | sigmoid | T3M1M0 | III |
| 195 | M | 65 | rectum | T4N0M0 | II |
| 196 | M | 76 | rectum | T3N0M0 | II |
| 198 | M | 59 | rectum | T4N1M0 | III |
| 199 | M | 73 | descending colon | T3N0M0 | II |
| 200 | M | 65 | sigmoid | T4N1M0 | III |
| 201 | M | 66 | sigmoid | T3N1M0 | III |
| 202 | F | 81 | transverse colon | T3N0M0 | II |
| 203 | F | 80 | rectum | T2N0M0 | I |

**Table S2** Differential metabolites between CRC and HC group

| **Variables** | **Compound ID** | **Description** | **Score** |
| --- | --- | --- | --- |
| 1.41_102.0553m/z | HMDB0061148 | Hydroxyethyl glycine | 42.9 |
| 0.67_280.0441n | HMDB0127482 | 6-[(2-carboxyacetyl)oxy]-3,4,5-trihydroxyoxane-2-carboxylic acid | 40.5 |
| 0.89_129.0662m/z | HMDB0000641 | L-Glutamine | 54.6 |
| 0.89_245.0751m/z | HMDB0030419 | gamma-Glutamylaspartic acid | 50 |
| 0.89_286.1103n | HMDB0038964 | 3-Hydroxy-4,6-heptadiyne-1-yl 1-glucoside | 47.7 |
| 0.91_275.1123n | HMDB0011738 | N2-gamma-Glutamylglutamine | 47.6 |
| 0.92_287.2445m/z | HMDB0002172 | N1,N12-Diacetylspermine | 51 |
| 0.94_273.1806n | METLIN23912 | Val Arg | 49.3 |
| 0.97_298.0783n | METLIN65112 | Ala-Thr-OH | 45.9 |
| 0.97_343.0497m/z | HMDB0125826 | [5-hydroxy-2-(hydroxymethyl)-7-methoxy-2-methyl-3,4-dihydro-2H-1-benzopyran-4-yl]oxidanesulfonic acid | 43 |
| 0.99_186.1065m/z | HMDB0060860 | OR-1855 | 36.6 |
| 0.99_229.0891n | METLIN53 | Ergothioneine | 54.5 |
| 1.01_134.0273m/z | HMDB0029432 | (S)C(S)S-S-Methylcysteine sulfoxide | 41.7 |
| 1.01_137.0327m/z | HMDB0000076 | Dihydrouracil | 44.2 |
| 1.01_291.0696m/z | HMDB0000425 | 3-Deoxy-D-glycero-D-galacto-2-nonulosonic acid | 52.9 |
| 1.02_232.1297m/z | HMDB0029122 | Valyl-Asparagine | 46.7 |
| 1.02_409.1327m/z | HMDB0128937 | 2-{9-hydroxy-2-oxo-2H,8H,9H-furo[2,3-h]chromen-8-yl}propan-2-yl (2E)-3-(4-hydroxyphenyl)prop-2-enoate | 47.7 |
| 3.63_281.0766m/z | HMDB0000884 | Ribothymidine | 47 |
| 1.05_188.1284m/z | HMDB0013010 | N-Heptanoylglycine | 47.2 |
| 1.05_304.1390n | METLIN17525 | Ala Ser Gln | 50.1 |
| 1.07_216.1113n | HMDB0029069 | Threoninyl-Proline | 50.3 |
| 1.07_246.1089m/z | METLIN91 | 2'-Deoxyuridine | 42.1 |
| 1.07_447.1612n | METLIN144902 | Gly Glu Asn Glu | 41.9 |
| 1.09_571.1858m/z | HMDB0130300 | 6-[2-(acetyloxy)-2-[6-(2-methylbut-3-en-2-yl)-7-oxo-2H,3H,7H-furo[3,2-g]chromen-2-yl]propoxy]-3,4,5-trihydroxyoxane-2-carboxylic acid | 51.9 |
| 1.12_292.1645n | HMDB0137256 | 3-(4-hydroxy-3-methoxyphenyl)-1-(3-pentyloxiran-2-yl)propan-1-one | 40.7 |
| 1.15_344.1019m/z | HMDB0006832 | S-Glutaryldihydrolipoamide | 40.9 |
| 1.16_202.1441m/z | HMDB0036998 | (S)-Oleuropeic acid | 42.1 |
| 1.16_222.0175m/z | HMDB0003484 | O-Phosphohomoserine | 44.5 |
| 1.16_261.0949m/z | HMDB0126546 | 3,4,5-trihydroxy-6-[(oxolan-2-yl)methoxy]oxane-2-carboxylic acid | 45.6 |
| 1.16_277.0689m/z | HMDB0000592 | Glucosamine 6-sulfate | 41.8 |
| 1.20_327.0918m/z | HMDB0127654 | 6-[2-carboxy-2,2-bis(hydroxymethyl)ethoxy]-3,4,5-trihydroxyoxane-2-carboxylic acid | 51.7 |
| 1.22_305.1098m/z | HMDB0061146 | Hydroxy-lacosamide | 42.5 |
| 1.23_261.1218n | HMDB0034777 | Epidermin | 46.3 |
| 1.23_281.1375n | HMDB0060699 | (E)-2-Hydroxy-N-desmethyldoxepin | 42.9 |
| 1.24_158.0930m/z | HMDB0000904 | Citrulline | 50.5 |
| 1.37_145.1340m/z | HMDB0002284 | N-Acetylcadaverine | 43.5 |
| 1.37_302.1218m/z | HMDB0011686 | p-Cresol glucuronide | 44.4 |
| 1.37_388.2314m/z | HMDB0036822 | (3S,7E,9S)-9-Hydroxy-4,7-megastigmadien-3-one 9-glucoside | 48.8 |
| 1.38_272.1015n | HMDB0004810 | 5C-aglycone | 45.2 |
| 1.38_288.2037m/z | METLIN23986 | Ile Arg | 54.4 |
| 1.43_146.0274m/z | METLIN66183 | 3,4-Dehydrothiomorpholine-3-carboxylate | 43.8 |
| 1.45_295.1176n | HMDB0029100 | Tyrosyl-Asparagine | 52.8 |
| 1.49_123.0556m/z | HMDB0002271 | Imidazolepropionic acid | 52.5 |
| 1.52_337.1896m/z | HMDB0035802 | S-Japonin | 35.8 |
| 1.52_359.1235n | HMDB0029452 | L-DOPA 3'-glucoside | 49.7 |
| 1.60_268.1064n | METLIN23814 | Tyr Ser | 49.6 |
| 1.75_309.1332n | HMDB0029119 | Tyrosyl-Gamma-glutamate | 51.1 |
| 1.83_233.0607m/z | HMDB0029901 | L-Galacto-2-heptulose | 44.5 |
| 1.84_207.0560m/z | HMDB0032414 | 2-Methyl-3 or 5 or 6-(furfurylthio)pyrazine (mixture of isomers) | 34.4 |
| 1.85_615.2121m/z | HMDB0006607 | 3'-Sialyllactosamine | 51.4 |
| 1.87_267.0593m/z | HMDB0000767 | Pseudouridine | 49.3 |
| 1.87_470.1156m/z | HMDB0125829 | 2-amino-4-[(2-{[2-carboxy-2-hydroxy-1-(4-hydroxyphenyl)ethyl]sulfanyl}-1-[(carboxymethyl)-C-hydroxycarbonimidoyl]ethyl)-C-hydroxycarbonimidoyl]butanoic acid | 44.6 |
| 1.96_213.1237m/z | METLIN23801 | Pro Pro | 45 |
| 1.98_288.2036m/z | METLIN24022 | Arg Leu | 52.8 |
| 2.12_170.0929m/z | HMDB0004194 | N1-Methyl-4-pyridone-3-carboxamide | 47 |
| 2.13_365.1748m/z | HMDB0033237 | 1,2,10-Trihydroxydihydro-trans-linalyl oxide 7-O-beta-D-glucopyranoside | 42.5 |
| 2.25_322.1880m/z | HMDB0028989 | Phenylalanyl-Arginine | 48.3 |
| 2.52_233.0362n | HMDB0004148 | Dopamine 4-sulfate | 53.1 |
| 2.58_338.1194m/z | HMDB0031182 | 1H-Indol-3-ylacetyl-myo-inositol | 48.1 |
| 2.59_316.1374m/z | HMDB0010350 | 2-Phenylethanol glucuronide | 49.9 |
| 2.69_152.0569m/z | HMDB0000403 | 2-Hydroxyadenine | 46.8 |
| 2.69_283.0923n | HMDB0000133 | Guanosine | 52 |
| 2.88_698.3100m/z | HMDB0038449 | Vomifoliol 9-[glucosyl-(1->4)-xylosyl-(1->6)-glucoside] | 38.2 |
| 2.91_698.3080n | HMDB0127587 | 3,4,5-trihydroxy-6-{[(16S)-5,7,11-trihydroxy-8,8,10,12-tetramethyl-3-[1-(2-methyl-1,3-thiazol-4-yl)prop-1-en-2-yl]-9-oxo-17-oxa-4-azabicyclo[14.1.0]heptadec-4-en-16-yl]methoxy}oxane-2-carboxylic acid | 52.5 |
| 2.92_458.2731m/z | METLIN244574 | Val Pro Arg Ser | 42 |
| 2.93_159.0769m/z | HMDB0029061 | Threoninyl-Glycine | 45.3 |
| 2.93_202.1323n | METLIN23864 | Ile Ala | 42 |
| 2.93_249.1965m/z | METLIN68567 | Matrine | 50.9 |
| 2.93_251.0804n | HMDB0029355 | N-Phenylacetylaspartic acid | 45.1 |
| 2.93_303.1459m/z | METLIN23702 | His Phe | 46.7 |
| 2.93_418.1941m/z | HMDB0030274 | (E)-Casimiroedine | 48.5 |
| 2.93_489.2317m/z | HMDB0129714 | 2-(2,4-dihydroxyphenyl)-5,7-dihydroxy-3-(8-hydroxy-3,7-dimethylocta-2,6-dien-1-yl)-6-(3-methylbut-2-en-1-yl)-4H-chromen-4-one | 40.4 |
| 2.93_820.8846n | METLIN832 | Iohexol | 43.3 |
| 2.94_267.1343m/z | METLIN23662 | Phe Thr | 49.5 |
| 2.94_341.1496n | HMDB0028896 | Histidinyl-Tryptophan | 52.4 |
| 2.94_367.1508m/z | HMDB0038690 | Isoachifolidiene | 53.2 |
| 2.94_401.2881m/z | METLIN20055 | Ile Ile Arg | 51.9 |
| 2.95_178.1341m/z | HMDB0000303 | Tryptamine | 45.5 |
| 2.95_340.1332m/z | HMDB0013200 | 5-Hydroxytryptophol glucuronide | 51.9 |
| 2.95_399.1764n | METLIN22330 | Phe Ser Phe | 47.1 |
| 2.96_944.4722m/z | HMDB0034870 | Spinacoside C | 45.2 |
| 2.97_645.3636m/z | HMDB0033729 | 1-Acetyl-3,27-dihydroxywitha-5,24-dienolide 3-glucoside | 42.8 |
| 2.99_202.1440m/z | HMDB0039052 | (4S,8R)-8,9-Dihydroxy-p-menth-1(6)-en-2-one | 44.8 |
| 2.99_335.1921m/z | HMDB0061077 | alpha-oxycodol | 40 |
| 2.99_905.4851m/z | HMDB0033500 | Lyciumoside VI | 44 |
| 3.00_389.1331m/z | HMDB0012492 | 1-(1,2,3,4,5-Pentahydroxypent-1-yl)-1,2,3,4-tetrahydro-beta-carboline-3-carboxylate | 52.6 |
| 3.00_467.1783m/z | HMDB0125915 | 6-{[2,2-dimethyl-6-(3-oxo-3-phenylpropyl)-2H-chromen-5-yl]oxy}-3,4,5-trihydroxyoxane-2-carboxylic acid | 43.1 |
| 3.00_684.3826m/z | HMDB0029335 | Mucronine D | 40.7 |
| 3.00_889.4908m/z | HMDB0036485 | Trigoneoside XIb | 45.2 |
| 3.01_241.1550m/z | HMDB0040686 | Isohumbertiol | 53.9 |
| 3.01_261.1314m/z | HMDB0003426 | Pantetheine | 43.9 |
| 3.01_280.1064n | METLIN64957 | Asp-Phe | 56.8 |
| 3.03_318.1918m/z | HMDB0030193 | N-2-[4-(3,3-Dimethylallyloxy)phenyl]ethylcinnamide | 44.2 |
| 3.03_894.4344m/z | HMDB0030010 | Glucoconvallatoxoloside | 43.5 |
| 3.03_943.2078m/z | HMDB0012155 | 3-Hydroxypimelyl-CoA | 46 |
| 3.04_181.0723m/z | HMDB0001889 | Theophylline | 49.1 |
| 3.05_236.1284m/z | HMDB0029040 | Serinyl-Hydroxyproline | 48.3 |
| 3.05_606.3315m/z | HMDB0004161 | D-Urobilin | 43.6 |
| 3.06_554.0472n | METLIN1624 | Ceftriaxone | 53.5 |
| 3.08_388.2215m/z | HMDB0002759 | Androsterone sulfate | 43.3 |
| 3.08_480.2139m/z | HMDB0035864 | 13-Hydroxy-5'-O-methylmelledonal | 49.2 |
| 3.09_245.1862m/z | HMDB0039358 | Ginsenoyne D | 41.3 |
| 3.09_255.1707m/z | STD208 | 17-beta-Estradiol | 44.9 |
| 3.09_462.2496m/z | HMDB0030156 | Austalide J | 50.1 |
| 3.10_280.1551m/z | HMDB0029437 | Nopalinic acid | 45.1 |
| 3.10_316.1912m/z | HMDB0030729 | Gravelliferone | 49.6 |
| 3.11_729.3516m/z | HMDB0030462 | Hordatine A glucoside | 36.1 |
| 3.11_767.5009m/z | HMDB0039545 | Ginsenoside F2 | 36.5 |
| 3.11_885.1916m/z | HMDB0001356 | 2-Methyl-3-hydroxybutyryl-CoA | 35.8 |
| 3.11_897.4310m/z | HMDB0038383 | 3b-Pregnadienolone 3-[rhamnosyl-(1->4)-rhamnosyl-(1->4)-rhamnosyl-(1->4)-glucoside] | 36.6 |
| 3.12_885.4229m/z | HMDB0060754 | 31-Hydroxy rifabutin | 48.1 |
| 3.13_223.1443m/z | HMDB0060678 | 3-Hydroxymonoethylglycinexylidide | 47.1 |
| 3.13_235.1807m/z | METLIN995 | Lidocaine | 44.7 |
| 3.13_313.1552m/z | METLIN23981 | Phe Phe | 49.4 |
| 3.13_315.1613m/z | HMDB0134713 | 3-{8-hydroxy-2-[(3E)-5-hydroxy-4-methylpent-3-en-1-yl]-2-methyl-2H-chromen-5-yl}propanoic acid | 46 |
| 3.13_402.1831m/z | HMDB0133003 | 3,4,5-trihydroxy-6-{[5-(4-methoxyphenyl)-3-oxopentyl]oxy}oxane-2-carboxylic acid | 48.7 |
| 3.13_869.2365n | HMDB0134935 | 6-{[2-(4-{[3-({3,4-dihydroxy-4-[(1H-indole-3-carbonyloxy)methyl]oxolan-2-yl}oxy)-4,5-dihydroxy-6-(hydroxymethyl)oxan-2-yl]oxy}phenyl)-4-oxo-3,4-dihydro-2H-1-benzopyran-7-yl]oxy}-3,4,5-trihydroxyoxane-2-carboxylic acid | 38.8 |
| 3.14_352.1662m/z | METLIN23919 | Phe Trp | 54.8 |
| 3.15_248.0921m/z | HMDB0039141 | Wyeronic acid | 45.8 |
| 3.15_264.1113n | HMDB0006344 | Phenylacetylglutamine | 53.8 |
| 3.15_456.0644m/z | METLIN533 | Cefotaxime | 54.8 |
| 3.15_980.6470m/z | HMDB0061572 | PS(DiMe(13,5)/DiMe(13,5)) | 43 |
| 3.17_243.1343m/z | HMDB0011170 | gamma-Glutamylisoleucine | 49.5 |
| 3.18_758.1958m/z | HMDB0125124 | ({6-[(2-{[2-(3,4-dihydroxyphenyl)-7-hydroxy-5-oxo-5H-chromen-3-yl]oxy}-4,5-dihydroxy-6-(hydroxymethyl)oxan-3-yl)oxy]-3,4,5-trihydroxyoxan-2-yl}methyl)[1-hydroxy-3-(4-hydroxyphenyl)prop-2-en-1-ylidene]oxidanium | 44.8 |
| 3.19_722.4317n | HMDB0029312 | Ophiopogonin C' | 44.6 |
| 3.19_784.4915m/z | HMDB0031357 | Pitheduloside A | 44.8 |
| 3.20_266.1269n | HMDB0139575 | (2E)-N-(4-amino-2,3-dihydroxybutyl)-3-(4-hydroxyphenyl)prop-2-enimidic acid | 45.7 |
| 3.20_303.1711m/z | HMDB0002171 | Glycylprolylhydroxyproline | 45.6 |
| 3.21_245.1499m/z | HMDB0035593 | Rishitin | 46.2 |
| 3.22_216.1597m/z | HMDB0013279 | N-Nonanoylglycine | 41.8 |
| 3.22_286.2168m/z | METLIN1751 | Pentazocine | 48.9 |
| 3.22_355.1230m/z | HMDB0004662 | S-(Hydroxymethyl)glutathione | 45.5 |
| 3.22_591.3186m/z | HMDB0002596 | Deoxycholic acid 3-glucuronide | 52.4 |
| 3.23_328.1120m/z | HMDB0031929 | Zanthodioline | 48.3 |
| 3.28_530.1334m/z | HMDB0127812 | 3,4,5-trihydroxy-6-{[3-hydroxy-2-(3-methoxyphenyl)-5-sulfino-3,4-dihydro-2H-1-benzopyran-7-yl]oxy}oxane-2-carboxylate | 46 |
| 3.29_165.0245m/z | HMDB0000469 | 5-(Hydroxymethyl)uracil | 48.4 |
| 3.29_346.1227m/z | METLIN1632 | Omeprazole | 57.1 |
| 3.30_430.3003m/z | METLIN162864 | Ile Lys Leu Gly | 49.1 |
| 3.30_603.2824m/z | METLIN73309 | Oxidized dinoflagellate luciferin | 43.9 |
| 3.30_823.5186m/z | HMDB0012373 | PS(16:1(9Z)/22:6(4Z,7Z,10Z,13Z,16Z,19Z)) | 44.1 |
| 3.32_331.2054m/z | HMDB0030259 | (+)-Erysotrine | 42.9 |
| 3.33_265.1054m/z | HMDB0013913 | 8-Hydroxynevirapine | 36 |
| 3.33_326.1967m/z | HMDB0036489 | Dihydrocumambrin A | 47 |
| 3.33_368.1862m/z | HMDB0036489 | Eremopetasin sulfoxide | 39.4 |
| 3.33_384.0461n | HMDB0128417 | 5-(acetyloxy)-14-hydroxy-9-oxo-8,17-dioxatetracyclo[8.7.0.0²,⁷.0¹¹,¹⁶]heptadeca-1(10),2(7),3,5,11,13,15-heptaen-13-yl 2-hydroxyacetate | 44.6 |
| 3.33_415.1695m/z | METLIN2268 | Diltiazem | 52.1 |
| 3.35_169.0432m/z | HMDB0029674 | 2-Chloro-1,3-dimethoxy-5-methylbenzene | 37 |
| 3.35_170.0516n | METLIN2177 | Propylthiouracil | 43.3 |
| 3.35_214.0414n | METLIN1233 | Baclofen b-(Chlorophenyl)-g-hydroxybutyric acidp- | 40.8 |
| 1.11_285.0439m/z | HMDB0140296 | 5,6,14-trihydroxy-8,17-dioxatetracyclo[8.7.0.02,?.011,1?]heptadeca-1(10),2(7),3,5,11(16),12,14-heptaen-9-one | 36.7 |
| 3.35_376.0970m/z | HMDB0029272 | 5,4'-Dihydroxy-3,3'-dimethoxy-6:7-methylenedioxyflavone | 44.9 |
| 3.35_466.0151m/z | HMDB0001201 | Guanosine diphosphate | 43.5 |
| 3.35_536.1822m/z | HMDB0125624 | 6-{[3,4-dihydroxy-5-(hydroxymethyl)-5-{[3,4,5-trihydroxy-6-(hydroxymethyl)oxan-2-yl]oxy}oxolan-2-yl]methoxy}-3,4,5-trihydroxyoxane-2-carboxylic acid | 53.9 |
| 3.36_138.0790m/z | HMDB0000699 | 1-methylnicotinamide | 48.9 |
| 3.36_139.0327m/z | METLIN45441 | 4-Pyridylthioamide | 44.8 |
| 3.36_156.0116m/z | METLIN1875 | 5-(2-Hydroxyethyl)thiazole-4-cabolactone | 46.4 |
| 3.36_463.1853n | HMDB0061137 | Dihydroisomorphine-6-glucuronide | 53.9 |
| 3.39_128.0458m/z | HMDB0060659 | 1-Methyl-4-nitroimidazole | 50.1 |
| 3.39_222.0457m/z | HMDB0030376 | (S)-Isowillardiine | 48 |
| 3.40_342.1740m/z | HMDB0032671 | (E)-2',4,4'-Trihydroxy-3-prenylchalcone | 43.3 |
| 3.40_382.2574n | HMDB0034953 | Apo-12'-violaxanthal | 42.2 |
| 3.41_470.3101n | HMDB0035113 | 17,23-Epoxy-29-hydroxy-27-norlanost-8-ene-3,15,24-trione | 42.3 |
| 3.42_235.0716m/z | HMDB0004086 | 5-Hydroxy-N-formylkynurenine | 48 |
| 3.42_325.2133m/z | HMDB0003598 | Retinyl ester | 47.8 |
| 3.44_362.0998m/z | HMDB0002666 | Thiamine monophosphate | 44.1 |
| 3.44_602.3863n | HMDB0033494 | Tetronasin | 40.4 |
| 3.52_517.1656n | METLIN1964 | Piperacillin | 44.8 |
| 3.52_608.2034m/z | HMDB0133930 | 2-amino-4-({1-[(carboxymethyl)-C-hydroxycarbonimidoyl]-2-[(2-hydroxy-5-oxo-1,7-diphenylhept-3-en-1-yl)sulfanyl]ethyl}-C-hydroxycarbonimidoyl)butanoic acid | 50.1 |
| 3.53_176.0708m/z | HMDB0060758 | 4-Anilino-4-oxobutanoic acid | 51.3 |
| 3.53_365.1353m/z | HMDB0133499 | 1-(4-hydroxy-3-methoxyphenyl)-7-(3-hydroxyphenyl)heptane-3,5-dione | 44.6 |
| 3.54_305.2481m/z | HMDB0036754 | Yucalexin P21 | 45.3 |
| 3.55_305.1767m/z | HMDB0032702 | Zeranol | 55.9 |
| 3.55_514.2381n | METLIN44651 | TELMISARTAN | 53.1 |
| 3.55_712.1815m/z | HMDB0128259 | [(3,4,5-trihydroxy-6-{1,2,6-trihydroxy-5-[hydroxy(3,4,5-trihydroxyoxan-2-yl)methyl]-3-[(2E)-3-(4-hydroxyphenyl)prop-2-enoyl]-4-oxocyclohex-2-en-1-yl}oxan-2-yl)methoxy]sulfonic acid | 40 |
| 3.56_430.3174m/z | HMDB0000712 | Hexadecanedioic acid mono-L-carnitine ester | 50.6 |
| 3.56_440.2993n | HMDB0012558 | 13'-Carboxy-gamma-tocotrienol | 40.3 |
| 3.57_389.2155m/z | HMDB0030597 | Umbelliprenin | 37.5 |
| 3.57_484.3256n | HMDB0037607 | Ganolucidic acid E | 42.4 |
| 3.57_590.4121m/z | HMDB0033078 | Ganoderic acid W | 42.9 |
| 3.58_454.3173m/z | HMDB0061047 | simvastatin hydroxy acid | 43.9 |
| 3.59_111.0807m/z | METLIN94803 | 2-Isopropylfuran | 45.4 |
| 3.60_468.2112m/z | HMDB0035062 | Arnamiol | 41.8 |
| 3.62_402.3144n | METLIN41986 | 25-hydroxy-23-oxavitamin D3 / 25-hydroxy-23-oxacholecalciferol | 44.4 |
| 3.63_613.3605m/z | HMDB0038377 | Goshonoside F4 | 43.1 |
| 3.66_324.1303m/z | METLIN63603 | Bialaphos | 42.4 |
| 3.73_314.1736n | HMDB0029848 | (-)-trans-Carveol glucoside | 46.4 |
| 3.75_420.2603m/z | HMDB0037531 | Dehydroxypaxilline | 46.4 |
| 3.79_369.0179n | METLIN2673 | H 151/72 | 39.2 |
| 3.80_268.2639m/z | HMDB0031336 | (Z)-9-Cycloheptadecen-1-one | 51.6 |
| 3.84_337.2385m/z | HMDB0001193 | 5(S)-Hydroperoxyeicosatetraenoic acid | 46.8 |
| 3.85_346.2150n | HMDB0006756 | 21-Hydroxy-5b-pregnane-3,11,20-trione | 49.5 |
| 3.87_304.2310m/z | STD732 | 4-Androstene-3,17-dione | 44.3 |
| 3.91_256.0971m/z | HMDB0029322 | 1-Hydroxy-3-methoxy-10-methylacridone | 51.1 |
| 3.92_201.0548m/z | HMDB0133611 | (2Z)-2-(hydroxymethyl)-3-phenylprop-2-enoic acid | 46.6 |
| 3.93_297.2672n | METLIN43208 | 3-ketosphingosine | 44.2 |
| 3.95_386.0790m/z | HMDB0006806 | Propinol adenylate | 42.6 |
| 4.00_328.1550m/z | HMDB0031744 | Artocarbene | 51 |
| 4.00_384.0408m/z | HMDB0133359 | [2,6-dihydroxy-3-(7-hydroxy-4-oxo-4H-chromen-2-yl)phenyl]oxidanesulfonic acid | 51 |
| 4.04_167.9938m/z | HMDB0030524 | 2(3H)-Benzothiazolethione | 48 |
| 4.06_298.3107m/z | METLIN64930 | Palmitoyl N-Isopropylamide | 47.7 |
| 4.09_361.2129m/z | HMDB0030270 | Anomurine | 47.8 |
| 4.10_311.0701m/z | HMDB0132663 | 2-(hydroxymethyl)-6-(2,4,6-trihydroxyphenyl)oxane-3,4,5-triol | 46 |
| 4.10_313.0674m/z | HMDB0140336 | 6-[(E)-2-(2H-1,3-benzodioxol-5-yl)ethenyl]-5-hydroxy-4-methoxy-5,6-dihydro-2H-pyran-2-one | 51 |
| 4.14_300.3262m/z | HMDB0001958 | Pristanal | 50.4 |
| 4.14_440.2752m/z | METLIN40154 | PC(O-6:0/6:0)[U] | 48.9 |
| 4.22_299.2828n | HMDB0000252 | Sphingosine | 51.5 |
| 4.25_596.4121m/z | HMDB0034194 | Jurubine | 45.2 |
| 4.29_272.1285m/z | HMDB0029374 | (E,E)-Trichostachine | 50.2 |
| 4.30_239.2370m/z | METLIN75364 | 2-hexadecenal | 51.9 |
| 4.30_298.3107m/z | HMDB0031810 | Tridemorph | 53.4 |
| 4.31_664.2394n | HMDB0129844 | 6-{[8-(2,4-dihydroxyphenyl)-7-(3,7-dimethylocta-2,6-dien-1-yl)-2,2-dimethyl-6-oxo-2H,6H-pyrano[3,2-g]chromen-5-yl]oxy}-3,4,5-trihydroxyoxane-2-carboxylic acid | 46.7 |
| 4.37_464.3337m/z | HMDB0030913 | Cepagenin | 47.9 |
| 4.45_625.4331m/z | HMDB0034977 | Karpoxanthin | 46.9 |
| 4.46_603.4184m/z | HMDB0036439 | Fasciculic acid A | 40.6 |
| 4.47_448.2339m/z | HMDB0038788 | Melleolide H | 47.9 |
| 4.50_301.1553m/z | HMDB0030191 | N-[2-Hydroxy-2-(4-hydroxyphenyl)ethyl]cinnamide | 52.8 |
| 4.52_306.1505n | HMDB0134982 | 2-(3,7-dimethylocta-2,6-dien-1-yl)-3,4,6-trihydroxybenzoic acid | 42.3 |
| 4.53_288.2458n | HMDB0011618 | All-trans-13,14-dihydroretinol | 49.9 |
| 4.57_322.0953m/z | HMDB0130177 | 5,6,7,8-tetrahydroxy-2-(4-hydroxyphenyl)-3,4-dihydro-2H-1-benzopyran-4-one | 43.3 |
| 4.65_565.2229n | HMDB0135505 | 6-[1-(3,4-dimethoxyphenyl)-2-{[3-(3,4-dimethoxyphenyl)-1-hydroxypropylidene]amino}ethoxy]-3,4,5-trihydroxyoxane-2-carboxylic acid | 47.2 |
| 4.65_676.4675m/z | HMDB0030492 | Spirolide B | 41.7 |
| 4.66_636.4201n | HMDB0039446 | Ginsenoside Rh7 | 44.9 |
| 4.70_614.4072n | HMDB0033335 | Antibiotic X 14889C | 48.6 |
| 4.75_171.0442m/z | METLIN2389 | 1-Hydroxy-2-naphthoic acid | 49.3 |
| 4.75_201.0548m/z | HMDB0031055 | (Z)-10-Hydroxy-8-decene-4,6-diynoic acid | 49.4 |
| 4.75_254.1389m/z | HMDB0011654 | 2-(3-Carboxy-3-(methylammonio)propyl)-L-histidine | 54.5 |
| 4.75_285.1369n | HMDB0029377 | Piperine | 57.1 |
| 4.75_288.1502m/z | HMDB0028715 | Arginyl-Methionine | 53.3 |
| 4.82_467.3021n | HMDB0010379 | LysoPC(14:0/0:0) | 54.9 |
| 4.82_496.2697m/z | HMDB0002639 | Sulfolithocholylglycine | 42.1 |
| 4.89_540.3072m/z | HMDB0010388 | LysoPC(18:3(9Z,12Z,15Z)) | 56.6 |
| 4.92_541.3178n | HMDB0010397 | LysoPC(20:5(5Z,8Z,11Z,14Z,17Z)) | 56.5 |
| 4.97_506.3253m/z | METLIN76569 | PC(17:2(9Z,12Z)/0:0) | 46.3 |
| 5.00_437.3744m/z | HMDB0006840 | 4,4-Dimethyl-5a-cholesta-8-en-3b-ol | 36.4 |
| 5.13_290.2252n | HMDB0005849 | 4-Androstenediol | 46.4 |
| 5.22_481.3178n | HMDB0010381 | LysoPC(15:0) | 53.1 |
| 5.24_312.1597m/z | HMDB0031693 | Ethyl (S)-3-hydroxybutyrate glucoside | 45.4 |
| 5.35_454.3899m/z | HMDB0000520 | 5a-Cholestane-3a,7a,12a,25-tetrol | 50.6 |
| 6.27_492.3638m/z | HMDB0035144 | (3beta,17alpha,23S,24S)-17,23-Epoxy-3,24,29-trihydroxy-27-norlanost-8-en-15-one | 38.3 |
| 6.41_496.3767m/z | METLIN43417 | 2-O-methyl PAF C-16 | 43.3 |

**Table S3** Differential metabolites for CRC distinction

| **Metabolites** | **AUC** | **Pval** | **Log_2_FC** |
| --- | --- | --- | --- |
| Guanosine | 0.951 | 3.54E-06 | 3.6752 |
| 2-Hydroxyadenine | 0.950 | 3.43E-07 | 2.6105 |
| Tyrosyl-Gamma-glutamate | 0.945 | 1.14E-10 | 2.9346 |
| Tyr Ser | 0.931 | 1.36E-10 | 2.3436 |
| Lyciumoside VI | 0.919 | 2.51E-05 | 3.7894 |
| 3-Hydroxypimelyl-CoA | 0.919 | 2.29E-11 | -2.9706 |
| N-Phenylacetylaspartic acid | 0.916 | 1.31E-10 | 1.4401 |
| Sphingosine | 0.914 | 1.70E-12 | -1.1486 |
| Val Arg | 0.908 | 1.32E-05 | 2.4348 |
| Phe Thr | 0.895 | 1.26E-08 | 1.2629 |
| LysoPC(14:0/0:0) | 0.892 | 1.31E-09 | -1.1188 |
| (S)C(S)S-S-Methylcysteine sulfoxide | 0.891 | 1.62E-11 | -1.136 |
| Ergothioneine | 0.882 | 4.99E-08 | -1.4554 |
| Tyrosyl-Asparagine | 0.877 | 3.65E-05 | 2.7267 |
| PC(17:2(9Z,12Z)/0:0)？ | 0.862 | 7.88E-06 | -1.0702 |
| OR-1855 | 0.858 | 1.72E-07 | -0.93552 |
| N-2-[4-(3,3-Dimethylallyloxy)phenyl]ethylcinnamide | 0.857 | 1.12E-05 | 2.33 |
| 3-ketosphingosine | 0.850 | 1.99E-07 | -1.0261 |
| Phenylalanyl-Arginine | 0.847 | 1.53E-06 | 3.9018 |
| Rishitin | 0.844 | 2.89E-06 | 2.2826 |
| Melleolide H | 0.843 | 3.29E-09 | -0.74221 |
| Valyl-Asparagine | 0.839 | 3.37E-03 | -4.2076 |
| N2-gamma-Glutamylglutamine | 0.838 | 9.13E-08 | 0.83288 |
| Imidazolepropionic acid | 0.838 | 3.01E-06 | -0.64202 |
| Vomifoliol 9-[glucosyl-(1->4)-xylosyl-(1->6)-glucoside] | 0.833 | 1.95E-07 | -0.99254 |
| PC(O-6:0/6:0)[U]？ | 0.831 | 8.09E-05 | -1.8207 |
| 3,4-Dehydrothiomorpholine-3-carboxylate？ | 0.830 | 2.42E-07 | -0.93032 |
| Mucronine D | 0.819 | 3.00E-06 | 1.4239 |
| Ribothymidine | 0.817 | 1.70E-06 | -0.94724 |
| Arg Leu | 0.817 | 7.13E-04 | 1.4457 |
| 5,6,7,8-tetrahydroxy-2-(4-hydroxyphenyl)-3,4-dihydro-2H-1-benzopyran-4-one | 0.816 | 1.53E-05 | -0.89101 |
| (2E)-N-(4-amino-2,3-dihydroxybutyl)-3-(4-hydroxyphenyl)prop-2-enimidic acid | 0.816 | 7.32E-04 | 2.3102 |
| PS(16:1(9Z)/22:6(4Z,7Z,10Z,13Z,16Z,19Z)) | 0.814 | 4.18E-06 | -2.3249 |
| 2-(2,4-dihydroxyphenyl)-5,7-dihydroxy-3-(8-hydroxy-3,7-dimethylocta-2,6-dien-1-yl)-6-(3-methylbut-2-en-1-yl)-4H-chromen-4-one | 0.814 | 2.47E-06 | 1.595 |
| D-Urobilin | 0.812 | 1.33E-05 | 1.0163 |
| O-Phosphohomoserine | 0.811 | 5.96E-03 | -1.9416 |
| Ginsenoyne D | 0.811 | 5.88E-03 | 1.1429 |
| Tetronasin | 0.811 | 3.40E-03 | 0.89187 |
| LysoPC(15:0) | 0.810 | 4.34E-06 | -0.70135 |
| Pitheduloside A | 0.805 | 2.24E-05 | -1.6392 |
| All-trans-13,14-dihydroretinol | 0.801 | 1.64E-03 | -1.995 |
| N1,N12-Diacetylspermine | 0.797 | 3.72E-06 | 1.7369 |
| (+)-Erysotrine | 0.797 | 8.54E-04 | -1.3239 |
| N1-Methyl-4-pyridone-3-carboxamide | 0.795 | 2.31E-06 | -0.60723 |
| 3,4,5-trihydroxy-6-[(oxolan-2-yl)methoxy]oxane-2-carboxylic acid | 0.794 | 1.04E-04 | 2.7228 |
| Piperine | 0.792 | 2.05E-03 | -2.8656 |
| (3S,7E,9S)-9-Hydroxy-4,7-megastigmadien-3-one 9-glucoside | 0.790 | 8.03E-03 | 1.1465 |
| Glucosamine 6-sulfate | 0.790 | 1.75E-04 | 3.081 |
| alpha-oxycodol | 0.789 | 6.83E-03 | 1.4659 |
| Val Pro Arg Ser | 0.787 | 2.95E-05 | 1.5011 |
| N-Heptanoylglycine | 0.783 | 3.32E-05 | -0.69611 |
| 3-Hydroxymonoethylglycinexylidide | 0.783 | 2.15E-05 | 0.63574 |
| (Z)-10-Hydroxy-8-decene-4,6-diynoic acid | 0.782 | 2.19E-03 | -2.9288 |
| Ala-Thr-OH | 0.778 | 7.95E-05 | -0.91129 |
| Pseudouridine | 0.778 | 6.19E-06 | -0.5928 |
| Ile Ala | 0.776 | 1.61E-04 | 0.70993 |
| (E,E)-Trichostachine | 0.776 | 1.63E-02 | -5.0866 |
| simvastatin hydroxy acid | 0.775 | 1.34E-05 | 1.3666 |
| Ethyl (S)-3-hydroxybutyrate glucoside | 0.772 | 2.42E-03 | -2.6598 |
| Asp-Phe | 0.772 | 1.35E-05 | 1.0225 |
| gamma-Glutamylisoleucine | 0.772 | 6.33E-03 | 1.5995 |
| Sulfolithocholylglycine | 0.772 | 6.74E-04 | -1.0864 |
| 1-Hydroxy-2-naphthoic acid | 0.769 | 3.10E-03 | -3.529 |
| (S)-Oleuropeic acid | 0.769 | 1.17E-05 | -0.85012 |
| Arginyl-Methionine | 0.768 | 2.59E-03 | -3.0746 |
| 3-Hydroxy-4,6-heptadiyne-1-yl 1-glucoside | 0.764 | 1.73E-02 | -2.3482 |
| 3-{8-hydroxy-2-[(3E)-5-hydroxy-4-methylpent-3-en-1-yl]-2-methyl-2H-chromen-5-yl}propanoic acid | 0.761 | 1.17E-05 | 0.84575 |
| (Z)-9-Cycloheptadecen-1-one | 0.760 | 6.83E-03 | -3.3001 |
| Glycylprolylhydroxyproline | 0.759 | 9.89E-03 | 1.7844 |
| Apo-12'-violaxanthal | 0.757 | 1.90E-03 | 0.67784 |
| 6-[(2-carboxyacetyl)oxy]-3,4,5-trihydroxyoxane-2-carboxylic acid | 0.755 | 2.10E-04 | -0.74991 |
| Phe Phe | 0.754 | 1.64E-05 | 0.78213 |
| 13'-Carboxy-gamma-tocotrienol | 0.754 | 2.54E-03 | 0.70973 |
| Dihydroisomorphine-6-glucuronide | 0.752 | 1.37E-02 | -1.7301 |
| Phe Trp | 0.747 | 7.95E-05 | 0.81284 |
| (2Z)-2-(hydroxymethyl)-3-phenylprop-2-enoic acid | 0.747 | 3.24E-02 | -1.528 |
| Hydroxyethyl glycine | 0.745 | 6.19E-04 | -0.75495 |
| Artocarbene | 0.745 | 2.81E-02 | -3.3682 |
| Histidinyl-Tryptophan | 0.743 | 2.96E-04 | 0.74578 |
| N-Nonanoylglycine | 0.742 | 9.49E-04 | 2.3434 |
| [5-hydroxy-2-(hydroxymethyl)-7-methoxy-2-methyl-3,4-dihydro-2H-1-benzopyran-4-yl]oxidanesulfonic acid | 0.742 | 3.22E-04 | -0.93535 |
| LysoPC(20:5(5Z,8Z,11Z,14Z,17Z)) | 0.742 | 5.40E-04 | -0.99755 |
| Pro Pro | 0.741 | 2.31E-03 | 0.98492 |
| Ile Ile Arg | 0.741 | 1.84E-05 | 1.9814 |
| Yucalexin P21 | 0.740 | 3.92E-02 | -0.99621 |
| ({6-[(2-{[2-(3,4-dihydroxyphenyl)-7-hydroxy-5-oxo-5H-chromen-3-yl]oxy}-4,5-dihydroxy-6-(hydroxymethyl)oxan-3-yl)oxy]-3,4,5-trihydroxyoxan-2-yl}methyl)[1-hydroxy-3-(4-hydroxyphenyl)prop-2-en-1-ylidene]oxidanium | 0.739 | 7.53E-06 | -2.1896 |
| 6-{[8-(2,4-dihydroxyphenyl)-7-(3,7-dimethylocta-2,6-dien-1-yl)-2,2-dimethyl-6-oxo-2H,6H-pyrano[3,2-g]chromen-5-yl]oxy}-3,4,5-trihydroxyoxane-2-carboxylic acid | 0.738 | 1.89E-03 | -0.61583 |
| 31-Hydroxy rifabutin | 0.736 | 1.03E-02 | 2.1265 |
| 2-O-methyl PAF C-16 | 0.733 | 1.14E-03 | -0.89395 |
| 1-Acetyl-3,27-dihydroxywitha-5,24-dienolide 3-glucoside | 0.732 | 3.13E-04 | 0.9953 |
| Ganolucidic acid E | 0.732 | 6.94E-03 | 0.67528 |
| Ile Lys Leu Gly | 0.732 | 2.20E-03 | 0.65123 |
| Theophylline | 0.731 | 3.36E-02 | -1.2088 |
| 17,23-Epoxy-29-hydroxy-27-norlanost-8-ene-3,15,24-trione | 0.730 | 8.59E-03 | 0.68564 |
| 3b-Pregnadienolone 3-[rhamnosyl-(1->4)-rhamnosyl-(1->4)-rhamnosyl-(1->4)-glucoside] | 0.727 | 1.70E-03 | 2.3566 |
| Anomurine | 0.726 | 4.94E-03 | 1.405 |
| S-Japonin | 0.725 | 7.17E-03 | 3.2373 |
| 6-[1-(3,4-dimethoxyphenyl)-2-{[3-(3,4-dimethoxyphenyl)-1-hydroxypropylidene]amino}ethoxy]-3,4,5-trihydroxyoxane-2-carboxylic acid | 0.723 | 1.53E-03 | -1.2528 |
| 17-beta-Estradiol | 0.721 | 1.35E-03 | 0.78781 |
| Hexadecanedioic acid mono-L-carnitine ester | 0.720 | 2.84E-03 | 0.89556 |
| 2'-Deoxyuridine | 0.719 | 4.17E-04 | 1.0622 |
| Deoxycholic acid 3-glucuronide | 0.718 | 2.88E-02 | 1.4042 |
| 6-{[2,2-dimethyl-6-(3-oxo-3-phenylpropyl)-2H-chromen-5-yl]oxy}-3,4,5-trihydroxyoxane-2-carboxylic acid | 0.714 | 1.00E-02 | 1.2284 |
| 1,2,10-Trihydroxydihydro-trans-linalyl oxide 7-O-beta-D-glucopyranoside | 0.713 | 1.37E-03 | -0.85714 |
| Ginsenoside F2 | 0.711 | 3.55E-02 | 1.9789 |
| Ala Ser Gln | 0.711 | 2.42E-04 | 0.86056 |
| Trigoneoside XIb | 0.710 | 4.40E-04 | 0.81749 |
| Serinyl-Hydroxyproline | 0.710 | 1.10E-02 | 1.2359 |
| 3-(4-hydroxy-3-methoxyphenyl)-1-(3-pentyloxiran-2-yl)propan-1-one | 0.709 | 1.48E-03 | 1.1988 |
| 21-Hydroxy-5b-pregnane-3,11,20-trione | 0.707 | 4.88E-02 | -0.98415 |
| 4-Androstene-3,17-dione | 0.706 | 2.27E-03 | -0.69904 |
| Glucoconvallatoxoloside | 0.706 | 1.26E-03 | -0.88565 |
| 2-amino-4-[(2-{[2-carboxy-2-hydroxy-1-(4-hydroxyphenyl)ethyl]sulfanyl}-1-[(carboxymethyl)-C-hydroxycarbonimidoyl]ethyl)-C-hydroxycarbonimidoyl]butanoic acid | 0.705 | 8.02E-04 | -0.62451 |
| Umbelliprenin | 0.703 | 2.28E-03 | 0.70492 |
| 1-Methyl-4-nitroimidazole | 0.703 | 4.95E-03 | -0.85321 |
| Ile Arg | 0.701 | 4.82E-03 | 0.72093 |
| 3,4,5-trihydroxy-6-{[3-hydroxy-2-(3-methoxyphenyl)-5-sulfino-3,4-dihydro-2H-1-benzopyran-7-yl]oxy}oxane-2-carboxylate | 0.701 | 3.40E-03 | 2.4232 |
| 25-hydroxy-23-oxavitamin D3 / 25-hydroxy-23-oxacholecalciferol | 0.701 | 2.70E-03 | 0.77408 |
| Ophiopogonin C' | 0.697 | 3.26E-02 | -0.91868 |
| Ganoderic acid W | 0.696 | 1.70E-02 | 0.74725 |
| (E)-Casimiroedine | 0.695 | 1.09E-03 | 0.67836 |
| Threoninyl-Proline | 0.694 | 3.04E-04 | 0.84162 |
| L-Galacto-2-heptulose | 0.694 | 1.66E-03 | 2.4861 |
| PS(DiMe(13,5)/DiMe(13,5)) | 0.694 | 4.86E-03 | -0.87222 |
| Gly Glu Asn Glu | 0.691 | 1.19E-03 | 0.7779 |
| Wyeronic acid | 0.687 | 1.07E-03 | 1.4534 |
| LysoPC(18:3(9Z,12Z,15Z)) | 0.687 | 1.73E-02 | -0.59182 |
| 1-(4-hydroxy-3-methoxyphenyl)-7-(3-hydroxyphenyl)heptane-3,5-dione | 0.684 | 2.35E-02 | -1.1054 |
| Pantetheine | 0.680 | 3.66E-02 | -0.6982 |
| 3,4,5-trihydroxy-6-{[(16S)-5,7,11-trihydroxy-8,8,10,12-tetramethyl-3-[1-(2-methyl-1,3-thiazol-4-yl)prop-1-en-2-yl]-9-oxo-17-oxa-4-azabicyclo[14.1.0]heptadec-4-en-16-yl]methoxy}oxane-2-carboxylic acid | 0.680 | 7.32E-03 | 0.72586 |
| Zeranol | 0.680 | 5.95E-03 | 0.79769 |
| Isohumbertiol | 0.679 | 2.62E-03 | 0.60386 |
| 5C-aglycone | 0.678 | 9.90E-03 | 1.2152 |
| Tryptamine | 0.678 | 1.84E-02 | -0.78066 |
| Phe Ser Phe | 0.675 | 1.19E-03 | -0.97682 |
| Bialaphos | 0.671 | 2.20E-02 | -1.0507 |
| Dopamine 4-sulfate | 0.669 | 2.98E-03 | -1.921 |
| 2-(hydroxymethyl)-6-(2,4,6-trihydroxyphenyl)oxane-3,4,5-triol | 0.668 | 3.77E-02 | -6.071 |
| Hordatine A glucoside | 0.667 | 2.39E-02 | 1.7532 |
| (4S,8R)-8,9-Dihydroxy-p-menth-1(6)-en-2-one | 0.666 | 1.03E-03 | 0.71144 |
| Androsterone sulfate | 0.666 | 3.35E-03 | 0.62039 |
| Goshonoside F4 | 0.666 | 2.34E-02 | 2.17 |
| His Phe | 0.665 | 5.67E-03 | 0.83503 |
| L-Glutamine | 0.664 | 8.21E-04 | 0.70536 |
| Antibiotic X 14889C | 0.664 | 2.15E-02 | 0.6496 |
| Spinacoside C | 0.663 | 2.21E-03 | -1.3268 |
| 13-Hydroxy-5'-O-methylmelledonal | 0.663 | 9.58E-03 | 0.82823 |
| Piperacillin | 0.661 | 3.40E-03 | -1.0941 |
| 5a-Cholestane-3a,7a,12a,25-tetrol | 0.659 | 6.21E-03 | 0.83447 |
| Ginsenoside Rh7 | 0.659 | 2.45E-02 | 0.61113 |
| L-DOPA 3'-glucoside | 0.657 | 3.64E-02 | -2.9056 |
| 1-(1,2,3,4,5-Pentahydroxypent-1-yl)-1,2,3,4-tetrahydro-beta-carboline-3-carboxylate | 0.655 | 3.43E-02 | 2.0641 |
| 2-Methyl-3-hydroxybutyryl-CoA | 0.654 | 3.25E-02 | 1.3482 |
| 5-Hydroxytryptophol glucuronide | 0.653 | 1.12E-02 | -0.88603 |
| 2-(3-Carboxy-3-(methylammonio)propyl)-L-histidine | 0.652 | 1.59E-02 | -0.72738 |
| 8-Hydroxynevirapine | 0.652 | 1.10E-02 | 0.74234 |
| Dehydroxypaxilline | 0.651 | 1.16E-02 | 1.0685 |
| 5(S)-Hydroperoxyeicosatetraenoic acid | 0.651 | 9.61E-03 | -0.92541 |
| 2-Methyl-3 or 5 or 6-(furfurylthio)pyrazine (mixture of isomers) | 0.650 | 3.24E-03 | 0.71681 |
| 4-Androstenediol | 0.650 | 4.64E-03 | 1.5317 |
| Phenylacetylglutamine | 0.650 | 4.80E-03 | 1.0037 |
| Spirolide B | 0.650 | 2.85E-02 | 0.59604 |
| Dihydrocumambrin A | 0.648 | 2.72E-02 | -0.98941 |
| (S)-Isowillardiine | 0.648 | 3.95E-02 | -1.4428 |
| Eremopetasin sulfoxide | 0.645 | 4.53E-02 | 0.92292 |
| Thiamine monophosphate | 0.645 | 1.77E-02 | 1.1159 |
| 6-[(E)-2-(2H-1,3-benzodioxol-5-yl)ethenyl]-5-hydroxy-4-methoxy-5,6-dihydro-2H-pyran-2-one | 0.644 | 4.26E-02 | -5.008 |
| Matrine | 0.642 | 8.33E-03 | 1.3571 |
| Fasciculic acid A | 0.641 | 4.44E-02 | 0.6484 |
| Propinol adenylate | 0.641 | 2.72E-02 | -1.2927 |
| 1-Hydroxy-3-methoxy-10-methylacridone | 0.638 | 1.15E-02 | -1.1027 |
| Isoachifolidiene | 0.637 | 1.18E-02 | 0.92938 |
| Karpoxanthin | 0.635 | 3.16E-02 | 0.64825 |
| (3beta,17alpha,23S,24S)-17,23-Epoxy-3,24,29-trihydroxy-27-norlanost-8-en-15-one | 0.635 | 3.19E-02 | -0.72506 |
| 6-[2-carboxy-2,2-bis(hydroxymethyl)ethoxy]-3,4,5-trihydroxyoxane-2-carboxylic acid | 0.634 | 8.40E-03 | -2.5207 |
| Hydroxy-lacosamide | 0.633 | 1.21E-02 | -2.2634 |
| Retinyl ester | 0.633 | 2.77E-02 | -0.60404 |
| (-)-trans-Carveol glucoside | 0.633 | 1.61E-02 | 1.0592 |
| 5-(2-Hydroxyethyl)thiazole-4-cabolactone | 0.631 | 2.15E-02 | 1.7941 |
| S-(Hydroxymethyl)glutathione | 0.631 | 7.98E-03 | 1.338 |
| 3,4,5-trihydroxy-6-{[5-(4-methoxyphenyl)-3-oxopentyl]oxy}oxane-2-carboxylic acid | 0.630 | 1.38E-02 | -0.94284 |
| Dihydrouracil | 0.629 | 1.83E-02 | 1.1921 |
| 5-Hydroxy-N-formylkynurenine | 0.629 | 1.32E-02 | -0.95603 |
| H 151/72 | 0.629 | 1.32E-02 | -0.95603 |
| [2,6-dihydroxy-3-(7-hydroxy-4-oxo-4H-chromen-2-yl)phenyl]oxidanesulfonic acid | 0.629 | 1.33E-02 | -0.95395 |
| Guanosine diphosphate | 0.628 | 9.87E-03 | 1.3287 |
| 2-amino-4-({1-[(carboxymethyl)-C-hydroxycarbonimidoyl]-2-[(2-hydroxy-5-oxo-1,7-diphenylhept-3-en-1-yl)sulfanyl]ethyl}-C-hydroxycarbonimidoyl)butanoic acid | 0.628 | 1.42E-02 | -0.94191 |
| Omeprazole | 0.626 | 1.47E-02 | 1.4306 |
| Austalide J | 0.623 | 1.82E-02 | 1.5991 |
| (E)-2',4,4'-Trihydroxy-3-prenylchalcone | 0.623 | 3.21E-02 | -0.86675 |
| Pentazocine | 0.620 | 9.55E-03 | 1.3151 |
| 6-{[3,4-dihydroxy-5-(hydroxymethyl)-5-{[3,4,5-trihydroxy-6-(hydroxymethyl)oxan-2-yl]oxy}oxolan-2-yl]methoxy}-3,4,5-trihydroxyoxane-2-carboxylic acid | 0.620 | 4.47E-02 | -0.76694 |
| Ceftriaxone | 0.619 | 1.94E-02 | -0.87843 |
| Nopalinic acid | 0.618 | 1.35E-02 | 1.3506 |
| 2-Phenylethanol glucuronide | 0.617 | 3.31E-02 | 1.2879 |
| Gravelliferone | 0.617 | 1.29E-02 | 1.2624 |
| [(3,4,5-trihydroxy-6-{1,2,6-trihydroxy-5-[hydroxy(3,4,5-trihydroxyoxan-2-yl)methyl]-3-[(2E)-3-(4-hydroxyphenyl)prop-2-enoyl]-4-oxocyclohex-2-en-1-yl}oxan-2-yl)methoxy]sulfonic acid | 0.616 | 2.77E-02 | 0.86133 |
| 2-(3,7-dimethylocta-2,6-dien-1-yl)-3,4,6-trihydroxybenzoic acid | 0.616 | 3.53E-02 | -0.66681 |
| S-Glutaryldihydrolipoamide | 0.615 | 4.36E-02 | -0.76248 |
| Lidocaine | 0.615 | 2.11E-02 | 1.107 |
| Zanthodioline | 0.615 | 1.47E-02 | 1.2887 |
| Diltiazem | 0.615 | 1.66E-02 | 1.1886 |
| 6-[2-(acetyloxy)-2-[6-(2-methylbut-3-en-2-yl)-7-oxo-2H,3H,7H-furo[3,2-g]chromen-2-yl]propoxy]-3,4,5-trihydroxyoxane-2-carboxylic acid | 0.614 | 1.85E-02 | 1.2034 |
| 3'-Sialyllactosamine | 0.614 | 1.85E-02 | 1.2034 |
| Cefotaxime | 0.614 | 1.85E-02 | 1.2034 |
| 5-(Hydroxymethyl)uracil | 0.614 | 1.85E-02 | 1.2034 |
| 2-Chloro-1,3-dimethoxy-5-methylbenzene | 0.614 | 1.80E-02 | 1.2052 |
| Propylthiouracil | 0.614 | 1.66E-02 | 1.1706 |
| Baclofen b-(Chlorophenyl)-g-hydroxybutyric acidp- | 0.614 | 1.84E-02 | 1.2037 |
| 5,6,14-trihydroxy-8,17-dioxatetracyclo[8.7.0.02,?.011,1?]heptadeca-1(10),2(7),3,5,11(16),12,14-heptaen-9-one | 0.614 | 1.85E-02 | 1.2034 |
| 1-methylnicotinamide | 0.614 | 1.85E-02 | 1.2034 |
| 4-Pyridylthioamide | 0.614 | 1.85E-02 | 1.2034 |
| 6-{[2-(4-{[3-({3,4-dihydroxy-4-[(1H-indole-3-carbonyloxy)methyl]oxolan-2-yl}oxy)-4,5-dihydroxy-6-(hydroxymethyl)oxan-2-yl]oxy}phenyl)-4-oxo-3,4-dihydro-2H-1-benzopyran-7-yl]oxy}-3,4,5-trihydroxyoxane-2-carboxylic acid | 0.613 | 2.20E-02 | 0.85634 |
| 5-(acetyloxy)-14-hydroxy-9-oxo-8,17-dioxatetracyclo[8.7.0.02,?.011,1?]heptadeca-1(10),2(7),3,5,11,13,15-heptaen-13-yl 2-hydroxyacetate | 0.613 | 2.09E-02 | 1.1046 |
| 1H-Indol-3-ylacetyl-myo-inositol | 0.612 | 2.28E-02 | 1.3478 |
| 2-{9-hydroxy-2-oxo-2H,8H,9H-furo[2,3-h]chromen-8-yl}propan-2-yl (2E)-3-(4-hydroxyphenyl)prop-2-enoate | 0.612 | 2.67E-02 | 1.1157 |
| TELMISARTAN | 0.612 | 2.09E-02 | 1.1625 |
| Iohexol | 0.610 | 4.48E-02 | 1.1294 |
| Oxidized dinoflagellate luciferin | 0.610 | 5.58E-03 | -2.861 |
| Cepagenin | 0.610 | 4.14E-02 | 0.67007 |
| 5,4'-Dihydroxy-3,3'-dimethoxy-6:7-methylenedioxyflavone | 0.609 | 4.68E-02 | -0.6787 |
| 2-hexadecenal | 0.606 | 2.69E-02 | 1.1169 |
| Threoninyl-Glycine | 0.606 | 3.57E-02 | 1.0199 |
| Pristanal | 0.605 | 2.27E-02 | 1.14 |
| Jurubine | 0.603 | 4.46E-02 | 0.70015 |
| Tridemorph | 0.601 | 3.36E-02 | 0.99505 |
| 2-Isopropylfuran | 0.600 | 1.54E-02 | 0.70006 |
| 4,4-Dimethyl-5a-cholesta-8-en-3b-ol | 0.598 | 6.34E-03 | -1.7737 |
| 3-Deoxy-D-glycero-D-galacto-2-nonulosonic acid | 0.596 | 1.48E-02 | 1.2138 |
| N-Acetylcadaverine | 0.596 | 3.76E-02 | 0.89743 |
| Citrulline | 0.593 | 1.82E-02 | -0.99776 |
| (E)-2-Hydroxy-N-desmethyldoxepin | 0.588 | 4.86E-02 | 1.083 |
| Palmitoyl N-Isopropylamide | 0.585 | 3.16E-02 | 1.0334 |
| Epidermin | 0.582 | 4.54E-02 | 0.90506 |
| 2(3H)-Benzothiazolethione | 0.582 | 2.68E-02 | 0.59853 |
| p-Cresol glucuronide | 0.580 | 4.96E-02 | 0.98021 |
| Arnamiol | 0.553 | 4.37E-02 | -0.87496 |
| gamma-Glutamylaspartic acid | 0.544 | 3.60E-02 | 0.9871 |
| N-[2-Hydroxy-2-(4-hydroxyphenyl)ethyl]cinnamide | 0.538 | 4.86E-02 | 2.1156 |
| 4-Anilino-4-oxobutanoic acid | 0.526 | 3.55E-02 | 0.65261 |

**Table S4** Comparison of differential metabolites in different groups

|  | Gender | | Tumor location | | AJCC stage | | TNM classification | | |
| --- | --- | --- | --- | --- | --- | --- | --- | --- | --- |
|  | Female  (n=22) | Male  (n=40) | Left  (n=50) | Right  (n=12) | I+II  (n=35) | III+IV  (n=27) | T  (n=35) | N  (n=24) | M  (n=3) |
|  | P | | P | | P | | P | | |
| Guanosine | 0.369 | | 0.715 | | 0.429 | | 0.420 | | |
| 2-Hydroxyadenine | 0.556 | | 0.675 | | 0.405 | | 0.567 | | |
| Tyrosyl-Gamma-glutamate | 0.814 | | 0.079 | | 0.757 | | 0.708 | | |
| Tyr Ser | 0.648 | | 0.624 | | 0.323 | | 0.439 | | |
| Lyciumoside VI | 0.444 | | 0.096 | | 0.898 | | 0.918 | | |
| 3-Hydroxypimelyl-CoA | 0.918 | | 0.195 | | 0.354 | | 0.619 | | |
| N-Phenylacetylaspartic acid | 0.722 | | 0.240 | | 0.128 | | 0.311 | | |
| Sphingosine | 0.149 | | 0.207 | | 0.484 | | 0.718 | | |
| Val Arg | 0.994 | | 0.005 | | 0.485 | | 0.656 | | |
