# Supplementary material for: LC–MS-based serum metabolomics analysis for the screening and monitoring of colorectal cancer
Source: Front Oncol. 2023 Jun 28;13:1173424. doi: 10.3389/fonc.2023.1173424 (PMC10338013; doi:10.3389/fonc.2023.1173424)

Figure S1 Assessment of QC samples.

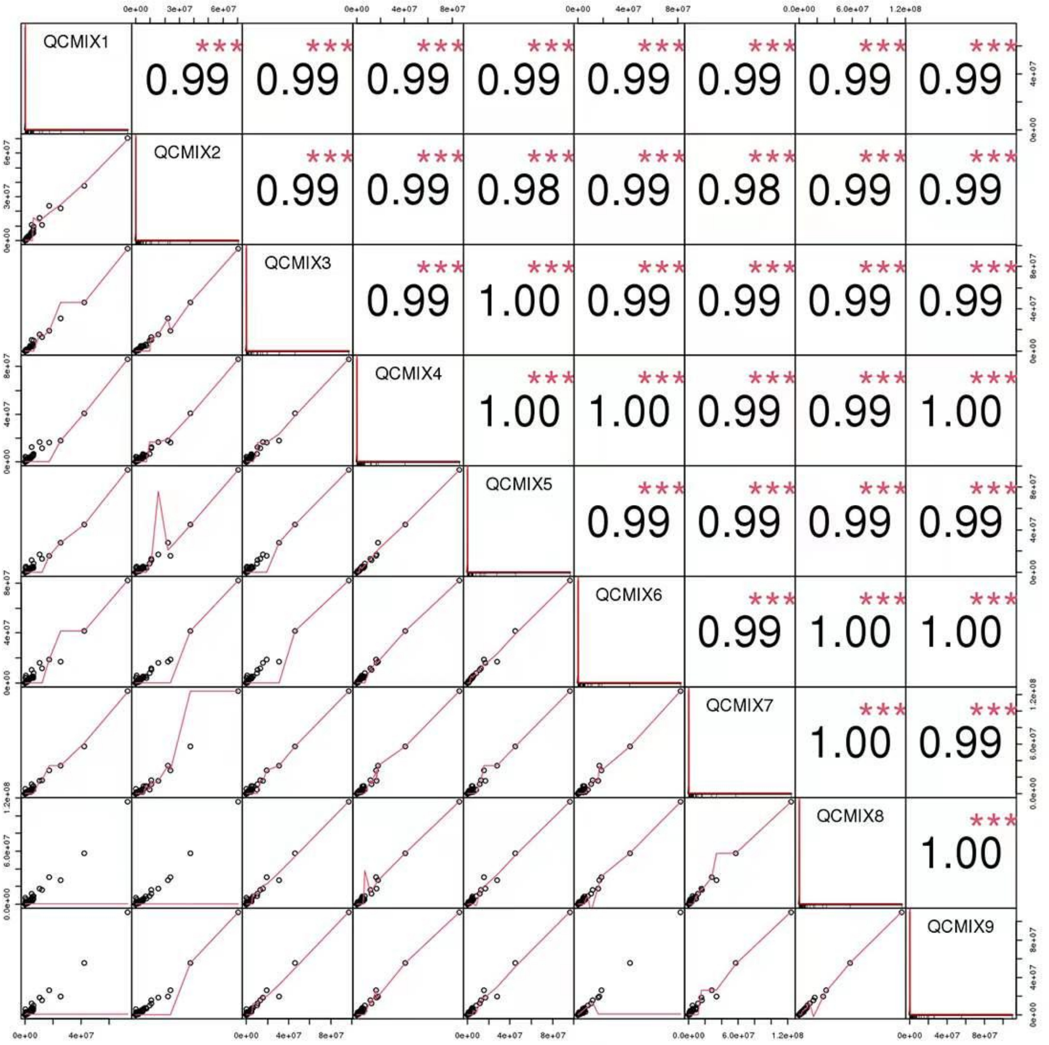

Figure S2 LC/MS serum chromatogram of QC sample

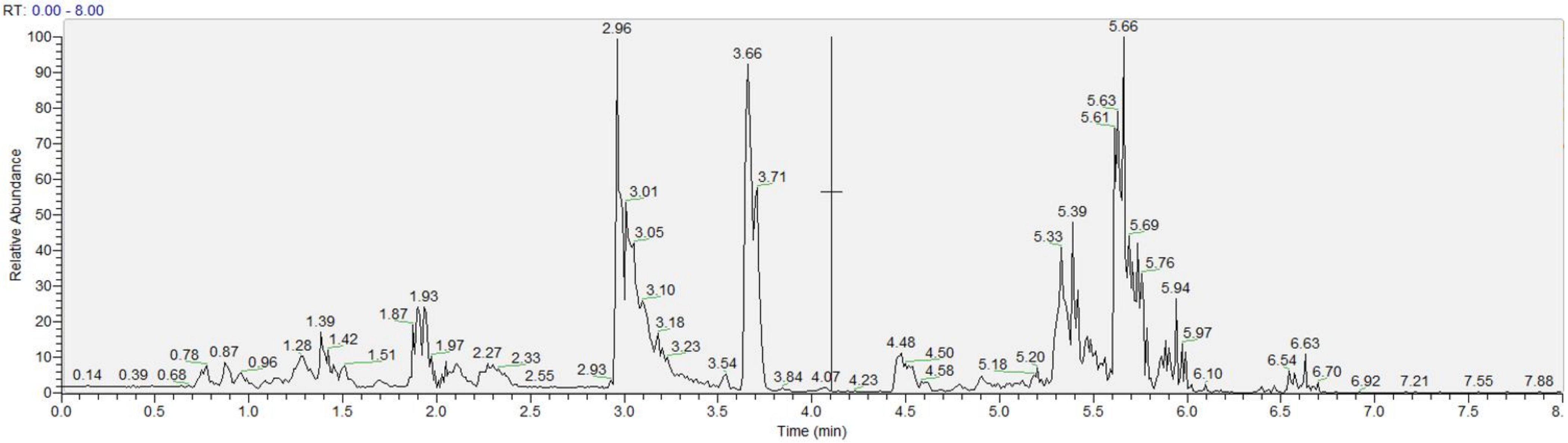

Figure S3 Metabolic profiling variation. **(a)** Score plot of the unsupervised PCA model among the CRC and HC. **(b)** One hundred permutation tests of the OPLS-DA model based on CRC and HC serum metabolome. R2=(0.0,0.815) Q2=(0.0,-0.421).

a

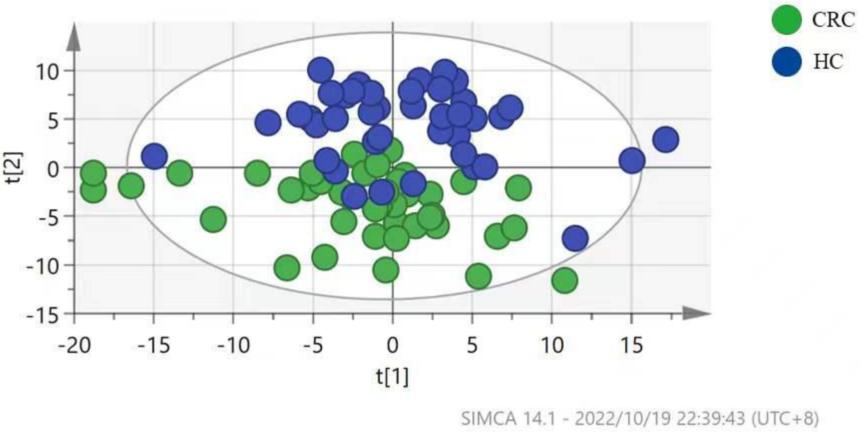

b

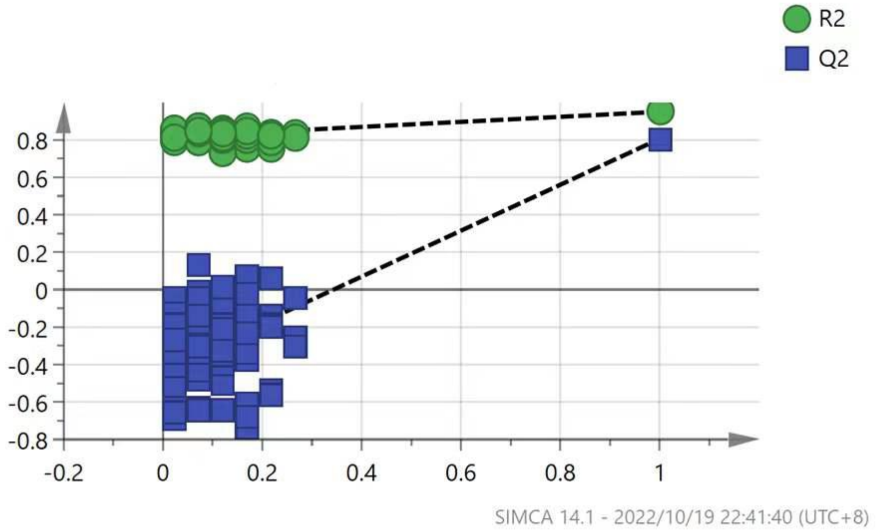

Figure S4 Mass spectra of 9 metabolites

Metabolite	Compound	Compound ID	Adducts	Formula	Score	Fragmentation Score
Guanosine	2.69_283.0923n	HMDB0000133	M+H, M+Na	C10H13N5O5	52	69.8

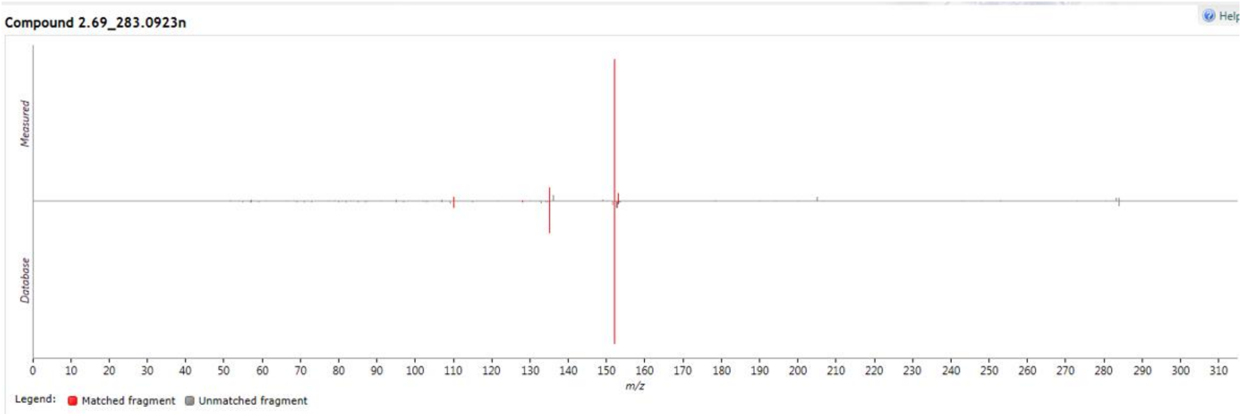

Metabolite	Compound	Compound ID	Adducts	Formula	Score	Fragmentation Score
2-Hydroxyadenine	2.69_152.0569m/z	HMDB0000403	M+H	C5H5N5O	42.7	18.6

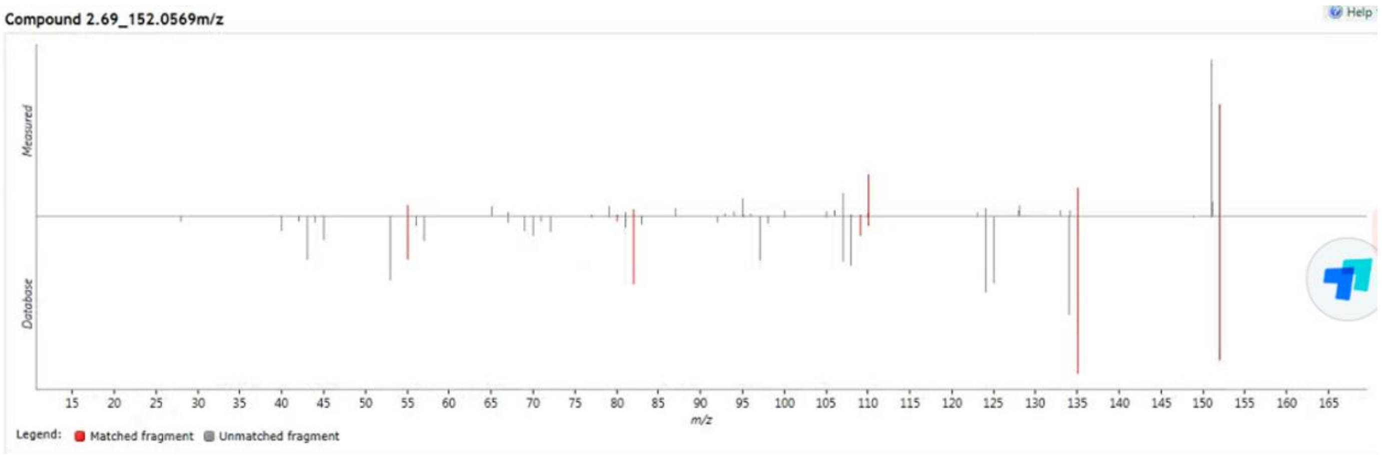

Metabolite	Compound	Compound ID	Adducts	Formula	Score	Fragmentation Score
Tyrosyl-Gamma-glutamate	1.75_309.1332n	HMDB0029119	M+H, M+Na	C14H19N3O5	43.3	26.7

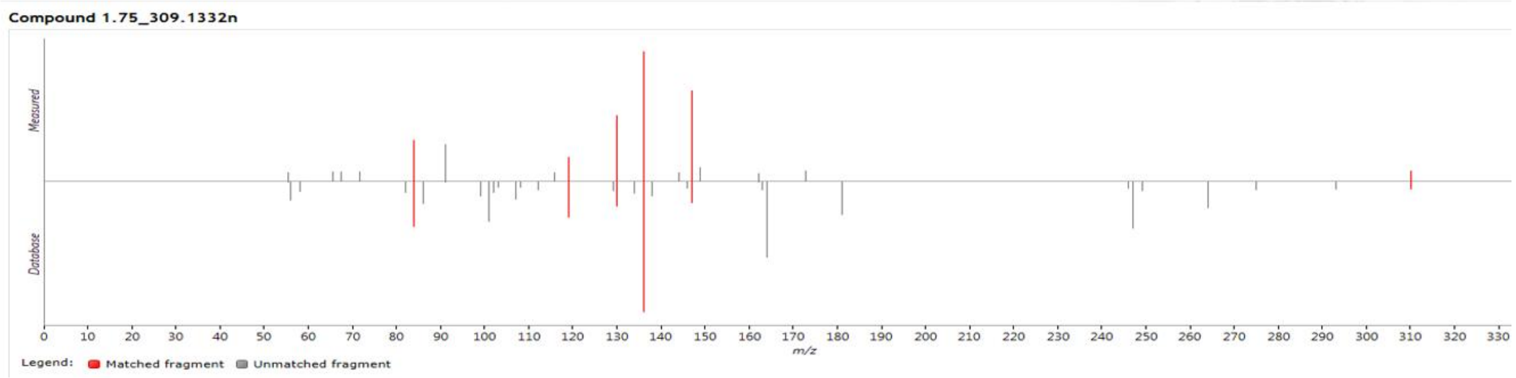

Metabolite	Compound	Compound ID	Adducts	Formula	Score	Fragmentation Score
Tyr Ser	1.60_268.1064n	METLIN23814	M+H, M+Na	C12H16N2O5	49.6	56

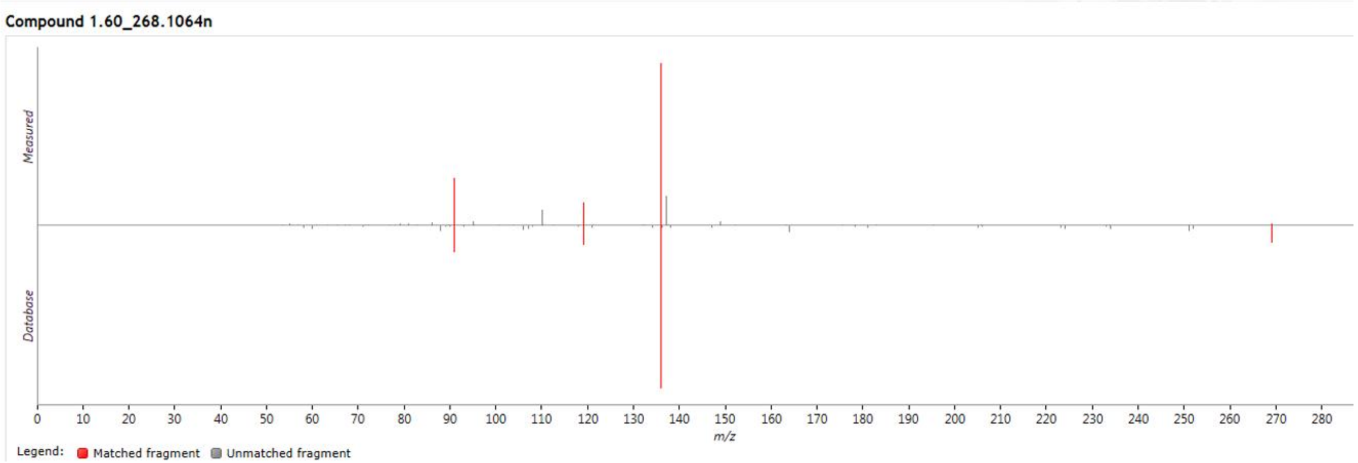

Metabolite	Compound	Compound ID	Adducts	Formula	Score	Fragmentation Score
Sphingosine	4.22_299.2828n	HMDB0000252	M+H-H2O, M+H	C18H37NO2	51.5	63.1

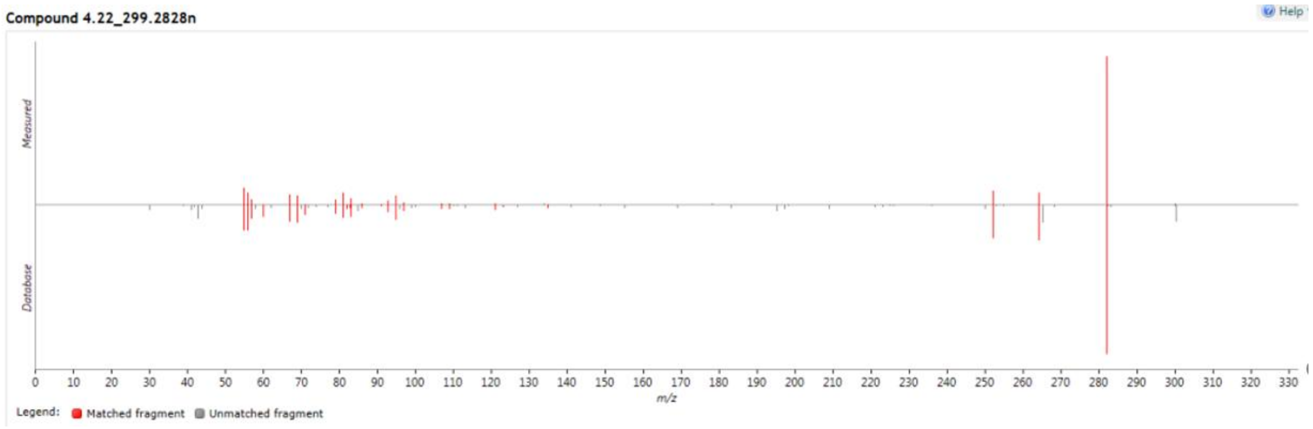

Metabolite	Compound	Compound ID	Adducts	Formula	Score	Fragmentation Score
Val Arg	0.94_273.1806n	METLIN23912	M+H, M+Na	$C_{11}H_{23}N_5O_3$	49.3	56

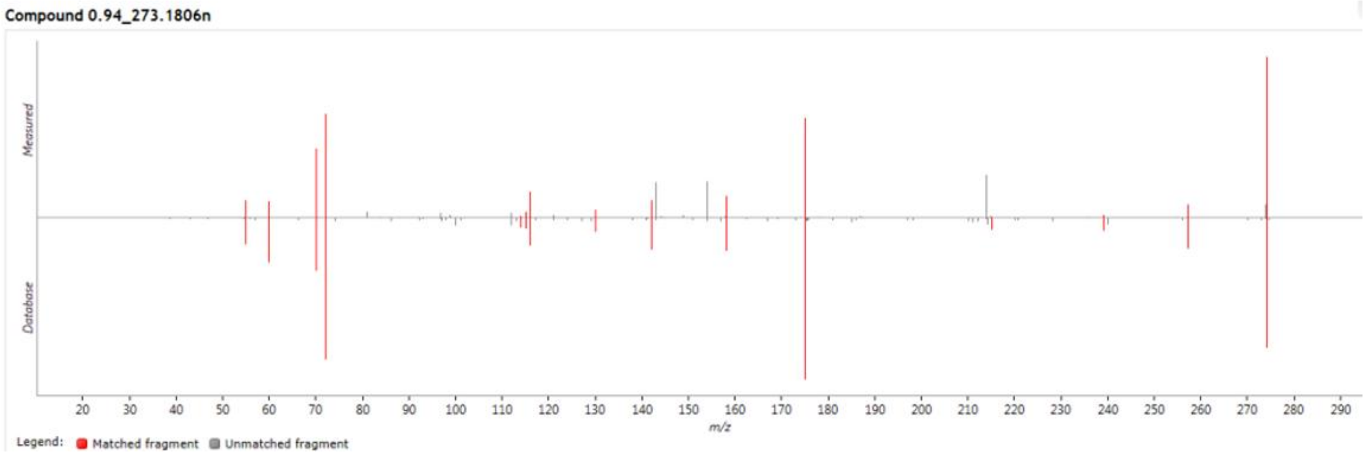

Metabolite	Compound	Compound ID	Adducts	Formula	Score	Fragmentation Score
Lyciumoside VI	2.99_905.4851m/z	HMDB0033500	M+H-H2O	C44H74O20	44	48.4

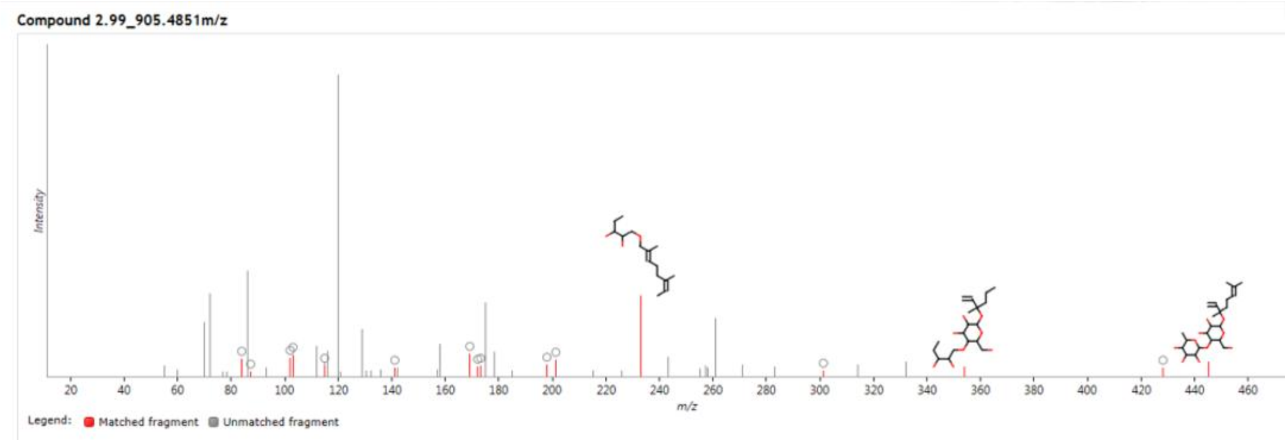

Metabolite	Compound	Compound ID	Adducts	Formula	Score	Fragmentation Score
3-Hydroxypimelyl-CoA	3.03_943.2078m/z	HMDB0012155	M+NH4	C28H46N7O20P3S	46	66.4

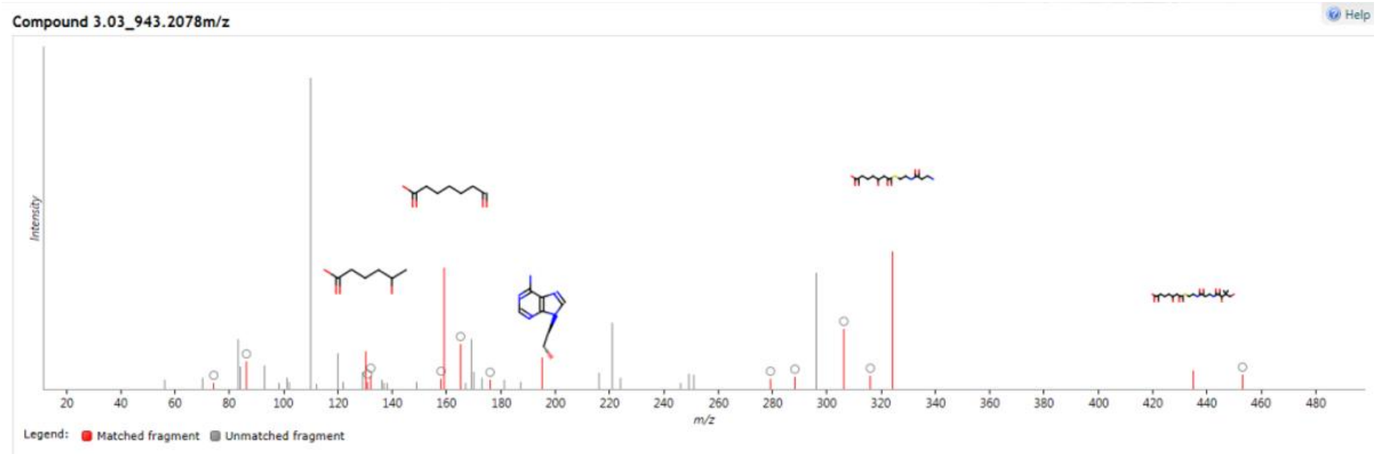

Metabolite	Compound	Compound ID	Adducts	Formula	Score	Fragmentation Score
N-Phenylacetylaspartic acid	2.93_251.0804n	HMDB0029355	M+H, M+NH4	C12H13NO5	45.1	34.5

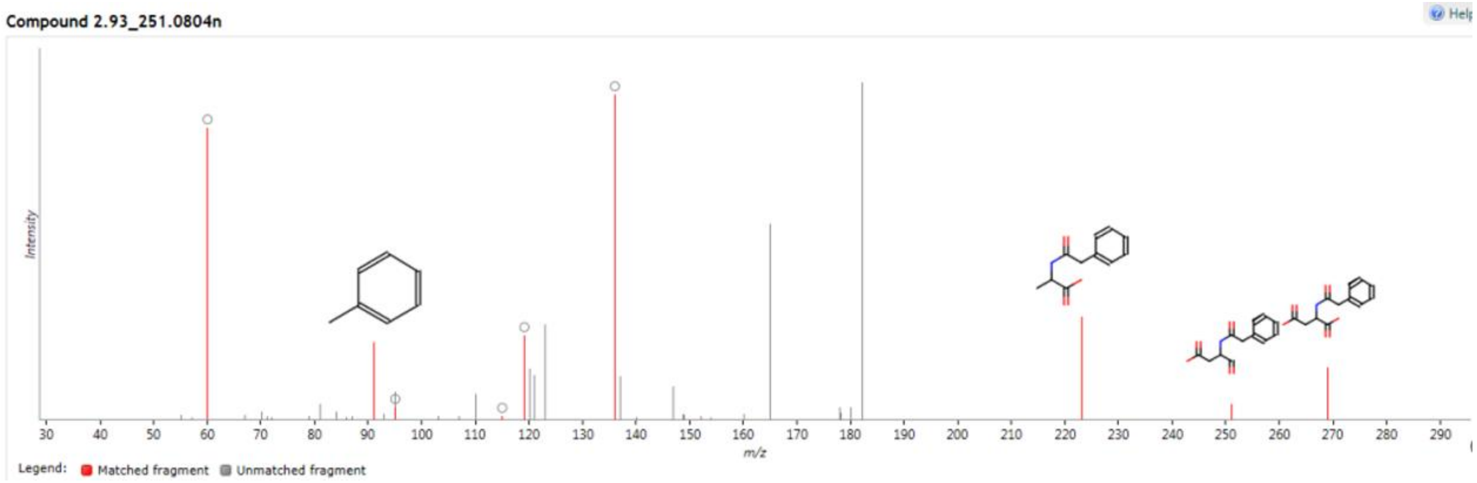

Figure S5 Box plots of 6 typical differential metabolites: **(a)** Guanosine, **(b)** 2-Hydroxyadenine, **(c)** Lyciumoside VI, **(d)** 3-Hydroxypimelyl-CoA, **(e)** Val Arg; "***"$p < 0.001$,"*"$p < 0.05$.

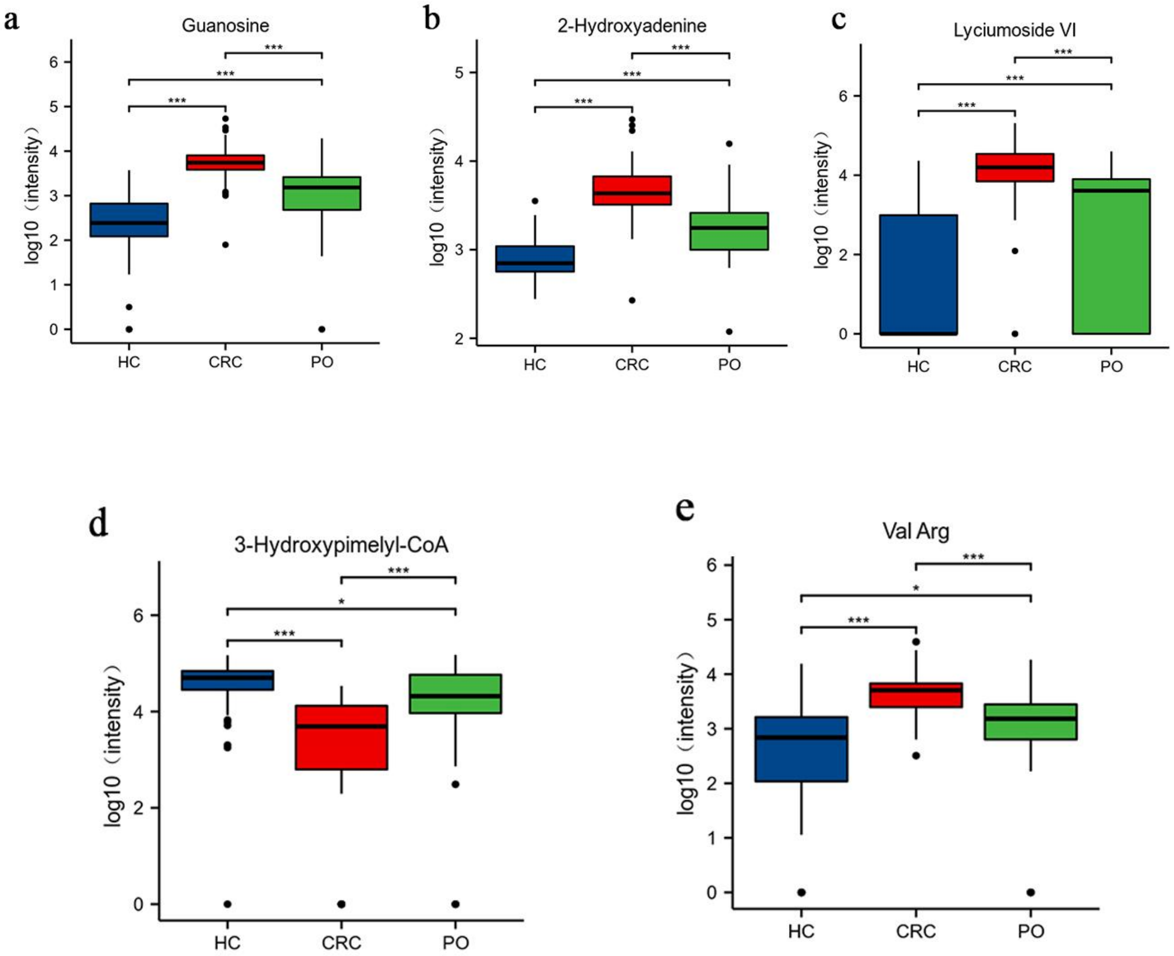

Supplement: Supplementary file 2 [file DataSheet_1.pdf]
